# Modulating medial septal cholinergic activity reduces medial entorhinal theta frequency without affecting speed or grid coding

**DOI:** 10.1038/s41598-017-15100-6

**Published:** 2017-11-06

**Authors:** Francis Carpenter, Neil Burgess, Caswell Barry

**Affiliations:** 1 Institute of Neurology, UCL, Queen Square, WC1N 3BG London, UK; 2 Research Department of Cell & Developmental Biology, UCL, Gower Street, WC1E 6BT London, UK; 3 Institute of Cognitive Neuroscience, UCL, Queen Square, WC1N 3AR London, UK

## Abstract

Medial septal inputs to the hippocampal system are crucial for aspects of temporal and spatial processing, such as theta oscillations and grid cell firing. However, the precise contributions of the medial septum’s cholinergic neurones to these functions remain unknown. Here, we recorded neuronal firing and local field potentials from the medial entorhinal cortex of freely foraging mice, while modulating the excitability of medial septal cholinergic neurones. Alteration of cholinergic activity produced a reduction in the frequency of theta oscillations, without affecting the slope of the non-linear theta frequency vs running speed relationship observed. Modifying septal cholinergic tone in this way also led mice to exhibit behaviours associated with novelty or anxiety. However, grid cell firing patterns were unaffected, concordant with an absence of change in the slopes of the theta frequency and firing rate speed signals thought to be used by grid cells.

## Introduction

The medial septal nucleus (MS) of the basal forebrain contains a mixture of GABAergic, glutamatergic, and cholinergic neurones, each with projections to the hippocampus and entorhinal cortex^1–4^. The MS is known to play a key role in the temporal processing of the hippocampal formation, acting as a ‘pacemaker’ of the 6–10 Hz theta oscillation seen throughout the region^5–11^. In addition, the MS is integral to animals’ spatial and navigational abilities^12,13^, perhaps due to its encoding of the animal’s running speed in the firing rates of specific neurones^14,15^. The precise roles of the diverse neural subtypes of the MS in these processes remain largely unknown however. In addition, one or both of these temporal and spatial functions of the MS may account for the observation that its inactivation disrupts the precise periodic firing patterns of grid cells^16,17^. Oscillatory interference models imply that the septum’s temporal processing is key, in proposing that velocity-dependent changes in the difference in frequency between two theta oscillations encode the animal’s speed and heading direction, allowing for the appropriate updating of grid cell firing^18^. Alternatively, continuous attractor network models hold that the septum’s spatial processing is paramount. Specifically, in suggesting that an animal’s running speed is encoded in the firing rates of septal and thus entorhinal neurones, which, when combined with directional information, can be used to shift the grid cell representation of self-location^19,20^. At present however, it is unknown which of these speed signals are used to update grid firing, and thus what the precise functional contribution of the MS to grid cell processing is.

Evidence for involvement of the MS in theta rhythmogenesis is compelling and longstanding, with lesions of the MS eliminating theta oscillations, and MS neurones bursting at theta frequencies^5–11^. Recent results consistently indicate a role for cholinergic neurones of the MS in movement-related theta oscillations, though one which remains largely elusive: Lesions of these neurones reduce theta power but do not abolish it^21,22^, optogenetically stimulating them in behaving mice spares theta frequencies while adjacent frequencies are attenuated^23^, and muscarinic receptor blockade abolishes the change in theta frequency with running speed^24^. However, because cholinergic neurones do not burst at theta frequencies, they are unlikely to be the ultimate ‘pacemakers’ of theta oscillations^25^. This function is generally attributed to the GABAergic MS neurones, which do burst at theta frequencies *in-vivo* ^25^, lead hippocampal theta^26^, and extensively contact hippocampal interneurons^27^, which in turn pace theta oscillations^28^. While glutamatergic MS neurones can also pace theta oscillations^14,29^, this depends on local connectivity within the MS, probably through connections onto GABAergic neurones^14^. That is, the exact involvement of MS cholinergic neurones in movement-related theta oscillations remains to be fully characterised.

The MS has also long been known to be central to animals’ spatial and navigational capacities: lesions of the MS prevent rats from learning to navigate to a goal purely by its location in a room^12^, and impair their ability to navigate to a hidden goal in the Morris water maze^13^. More recent results suggest that the MS may contribute to spatial processing through encoding the animal’s running speed in the firing rates of specific neurones. For example, inactivation of the MS impairs animals’ ability to estimate linear distances travelled^30^, abolishes the distance-specific firing of place cells on a running wheel^31^, and modulates the speed-rate coding of entorhinal neurones^32^. Further, inactivation of the MS disrupts the periodic spatial firing of grid cells without affecting their directional tuning^16,17^. Indeed, firing rate coding of running speed has been observed in glutamatergic neurones of the MS^14^, as well as their entorhinal-projecting axons^15^. However, the extent to which cholinergic MS neurones are involved in the encoding of running-speed is unknown.

Given this context of uncertainty regarding the involvement of cholinergic neurones in the septum’s generation of theta oscillations and its firing rate code for running speed, we recorded single units and local field potentials from the medial entorhinal cortex of foraging mice while modulating the excitability of MS cholinergic neurones using Designer Receptors Exclusively Activated by Designer Drugs (DREADDs). As well as aiming to shed light on the role of cholinergic neurones in these septal functions, we hoped also to gain insight into the relative involvement of theta frequency and firing rate speed signals in the generation of grid cell firing patterns. If modulating MS cholinergic activity altered the difference between the constituent theta oscillators in how their frequencies varied with running speed, oscillatory interference models predict that the scale of the grid pattern should change^18^. Indeed, it is this mechanism by which increases in acetylcholine concentration in novel contexts have been suggested to give rise to the increase in grid scale observed when rats are first exposed to a novel environment^33,34^. Alternatively, continuous attractor network models predict that the scale of the grid pattern should vary if modulating MS cholinergic activity altered the slope of the firing rate vs running speed relationship seen in entorhinal neurones^19,20^. While using DREADDs to modulate MS cholinergic activity reduced the frequency of theta oscillations, there was no change in the slope of the non-linear theta frequency vs running speed relationship observed, nor in the phase precession exhibited by entorhinal grid cells. Similarly, no change was observed in the relationship between firing rate and running speed in recorded grid cells. The absence of change in the slopes of these putative speed signals was consistent with a lack of change in grid cell firing patterns. These results therefore provide evidence for an involvement of MS cholinergic neurones in the determination of the frequency of theta oscillations, but not in the determination of theta-frequency and firing-rate speed signals, preventing conclusions being drawn regarding which is central to the updating of the grid cell code for self-location. Interestingly, modulation of septal cholinergic activity also led mice to display a pattern of behaviours consistent with anxiety or the detection of novelty.

## Results

**Figure 1:**
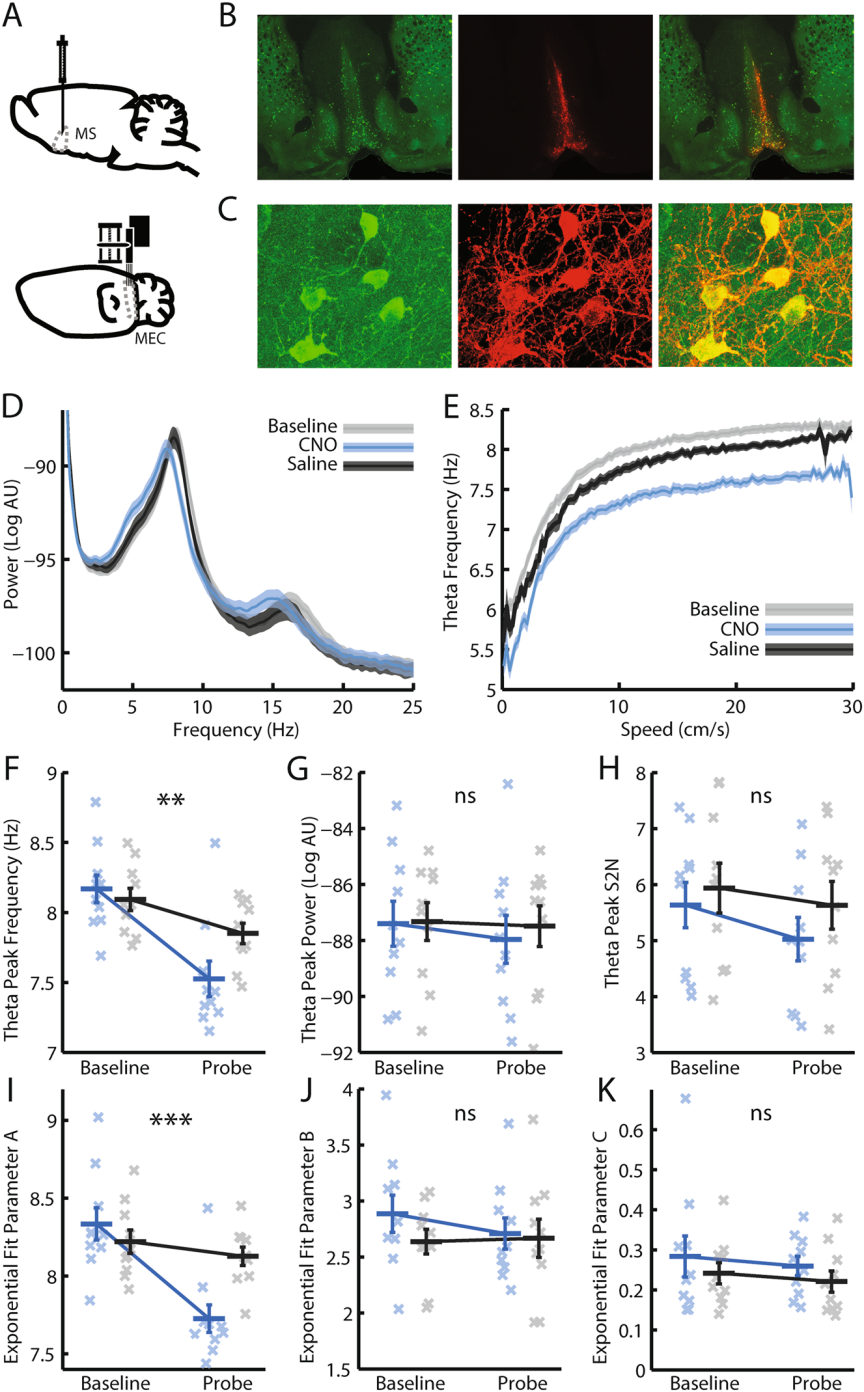
Modulating medial septal cholinergic activity reduces LFP theta frequency by shifting its relationship with running speed to lower frequencies. (**A**) Schematic representation of the experiment design: the Cre-dependent excitatory DREADD hM3Dq was injected into the medial septum of ChAT-Cre mice. In the same animal, tetrodes were implanted in the medial entorhinal cortex. (**B**) 2.5x epifluorescence images of coronal brain sections showing ChAT expression (green) and mCherry expression (red), with expression of the latter limited to the medial septum. (**C**) 40x confocal z-stack images demonstrating tight overlap between ChAT and mCherry expression. (**D**) Average LFP power spectra: mean ± SEM log power at each frequency across all baseline, CNO, and saline probe trials respectively. (**E**) Mean ± SEM frequency of maximum power in the 3–11 Hz range at each running speed, averaged across all baseline, CNO, and saline probe trials respectively. (**F**–**K**) Blue = CNO, black = saline. Bars indicate mean ± SEM across all animals in each group. Crosses indicate average value for each animal in each group. Stars indicate RM-ANOVA baseline vs probe * CNO vs saline interaction significance. ***p < 0.001, **p < 0.01, *p < 0.05, ns = p > 0.05. (**F**) Frequency of maximum power in the LFP power spectrum in the theta (6–10 Hz) band. (**G**) Maximum log power in the theta band of the LFP power spectrum. (**H**) Theta S2N: the ratio of average power in a 2 Hz window around the theta peak compared to average power in the rest of the 3–25 Hz range. (**I**–**K**) Three-term exponential fit parameter A, B, and C values respectively that best fitted the LFP theta frequency vs running speed relationship.

To investigate the function of MS cholinergic neurones, we modulated their excitability using DREADDs, while recording extracellularly from grid cells in medial entorhinal cortex (Fig. 1A). The Cre-dependent AAV2-hSyn-DIO-hM3D(Gq)-mCherry virus was injected into the MS of ChAT-IRES-Cre mice, so as to express hM3Dq exclusively in cholinergic neurones of the MS. Expression of hM3Dq (a modified G*q*-coupled human M3 muscarinic receptor) leads subsequent intraperitoneal injection of Clozapine-N-Oxide (CNO), to increase the excitability of hM3Dq expressing neurones^35^, having first been converted to Clozapine^36^. As such, administration of CNO may be expected to elevate septal cholinergic activity. However, without direct recordings from these neurones, and given local connections from cholinergic onto GABAergic neurones within the septum^37^, we cannot conclusively state whether the DREADD manipulation ultimately resulted in a net increase or decrease in septal cholinergic tone, and thus simply refer to the manipulation as a modulation of cholinergic activity (see *Discussion* for further consideration of this issue). Immunohistochemical staining for Choline Acetyltransferase (ChAT) and mCherry confirmed that expression of hM3Dq was limited to the MS (Fig. 1B), while confocal imaging indicated a tight overlap in expression of ChAT and mCherry (Fig. 1C). Cell counting revealed that mCherry was expressed in the majority of ChAT + neurones of the MS (Supp Fig. 1D; mean ± SEM proportion of ChAT + neurones co-labelled for mCherry = 0.57 ± 0.039), while almost all mCherry + neurones were ChAT + (Supp Fig. 1E; mean ± SEM proportion of mCherry + neurones co-labelled for ChAT = 0.95 ± 0.0032), indicating that hM3Dq was expressed almost exclusively in cholinergic neurones.

Electrophysiological recordings were made from the medial entorhinal cortex of 10 mice exploring a familiar environment during ‘baseline’ trials and one hour after injection of either CNO (3 mg/kg) or an equivalent volume of saline (‘CNO probe’ and ‘saline probe’ trials respectively; see Supp Fig. 1E for full experimental protocol). Following injection of CNO, a clear reduction in the frequency of theta oscillations was observed (Fig. 1D), apparent in speed-matched LFP traces from baseline and CNO probe trials (Supp Fig. 1F). Repeated Measures Analysis of Variance (RM-ANOVA) was used to assess whether there was a significant difference between CNO and saline injections in the change in theta frequency between baseline and probe trials. Indeed, CNO significantly reduced the frequency of theta oscillations (Fig. 1F; RM-ANOVA, trial*drug interaction; *F*_*(1,9)*_ = 15.50, *p* = 0.003), but did not affect their absolute power, nor their ‘signal to noise’ (S2N: ratio of average power in a 2 Hz band centred on the theta peak compared to average power in the rest of the 3–25 Hz range) (Fig. 1G,H; RM-ANOVA, trial*drug interaction; log power: *F*_*(1,9)*_ = 0.39, *p* = 0.25; S2N: *F*_(*1,9*)_ = 0.23, *p* = 0.37). Hence, analysis of power spectra indicated that modulating the activity of MS cholinergic neurones in this way reduced the frequency of LFP theta oscillations without changing their power.

The frequency of theta oscillations vary as a function of the animal’s running speed^38,39^. We therefore analysed what impact the DREADD manipulation of cholinergic activity had on the theta frequency vs running speed relationship. Wavelets were used to generate instantaneous estimates of the LFP power spectrum, which were combined to provide average power spectra in running speed bins of width 0.25 cm/s from 0–30 cm/s. For each speed bin the frequency of maximum power in a broad theta-band (3–11 Hz) was identified. The theta frequency vs running speed relationship consistently appeared to be non-linear (Fig. 1E, Supp Fig. 2A). Analysis of artificial LFP traces confirmed this was not an artefact of the analysis procedure (Supp Fig. 2B). The data were therefore fit with both linear (*y* = *A* + *B* * *x*) and three-term exponential (*y* = *A* − *B* ∗ *e* ^[−*C* ∗ *x*]^) functions using linear and non-linear least squares respectively, and the F-test used to assess whether there was a significant difference in the quality of the fits. In 100% of the 95 baseline, CNO, and saline probe trials performed, the exponential fit was significantly better (all p < 0.001); subsequent analyses of theta frequency vs running speed assume the non-linear fit. Following injection of CNO, the average theta frequency vs running speed relationship was shifted to lower frequencies (Fig. 1E). This change was reflected in a significant reduction in the parameter A value which best fitted the theta frequency vs running speed relationship (Fig. 1I; RM-ANOVA, trial*drug interaction; *F*_*(1,9)*_ = 37.42, *p* = 0.00018). In contrast, there was no significant effect of CNO on Parameter B or C (Fig. 1J,K; RM-ANOVA, trial*drug interaction; Parameter B: *F*_*(1,9)*_ = 1.15, *p* = 0.31; parameter C: *F*_*(1,9)*_ = 0.0044, *p* = 0.95). Alteration of the excitability of MS cholinergic neurones therefore decreased the frequency of theta oscillations by reducing the average frequency observed at each running speed, without affecting the shape of the non-linear relationship between the animal’s running speed and theta frequency.

**Figure 2:**
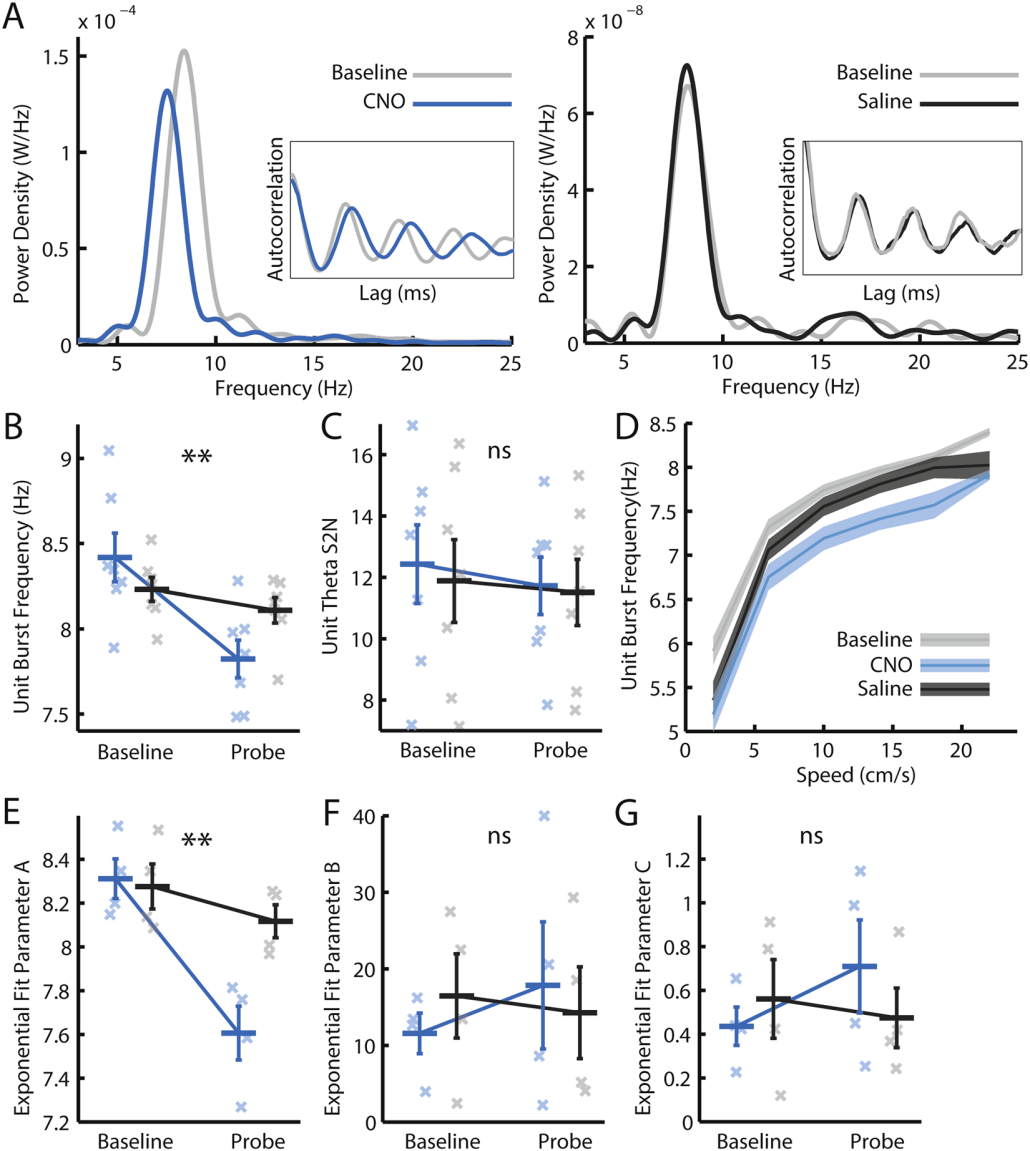
Modulating medial septal cholinergic activity reduces the burst frequency of theta-modulated non-spatial cells. (**A**) Example power spectra indicating the power at each frequency in the 3–25 Hz range in temporal autocorrelograms of spike trains from two theta-modulated single units. *Left*: a unit in a baseline trial and the same unit in a CNO probe trial. *Right:* a unit in a baseline trial and the same unit in a saline probe trial. *Inset*: smoothed spike train temporal autocorrelograms from the same cells/trials from which the power spectra were derived. (**B**,**C**,**E**–**G**) Blue = CNO, black = saline. Bars indicate mean ± SEM across all animals in each group. Crosses indicate average value for each animal in each group. Stars indicate RM-ANOVA baseline vs probe * CNO vs saline interaction significance. ***p < 0.001, **p < 0.01, *p < 0.05, ns = p > 0.05. (**B**,**C**) Only cells with significant theta modulation (assessed by a shuffle procedure) in either a baseline or probe trial are included. (**B**) Theta burst frequency of single units, as inferred from the frequency of maximum power in the 6–10 Hz theta window of each unit’s power spectrum. (**C**) Theta S2N: the ratio of average power in a 2 Hz window around the theta peak compared to average power in the rest of the 3–25 Hz. (**D**) Mean ± SEM unit burst frequency of maximum power in the 3–11 Hz range in each running speed bin, averaged across all baseline, CNO, and saline probe trials with significant theta modulation respectively. (**E**–**G**) Parameter A, B, and C values respectively that best fit the single unit theta frequency vs running speed relationship. Only cells with significant theta modulation (assessed by a shuffle procedure) in either a baseline or probe trial, and only trials with a significantly better exponential than linear fit are included.

Theta oscillations can also be observed in the bursts of action potentials emitted by individual neurones of the medial entorhinal cortex^40^. We therefore asked whether modulating the excitability of MS cholinergic neurones affected the theta bursting of single units. The theta-band modulation of cells’ firing was quantified from the power spectra of the temporal autocorrelogram of each neurone’s spike train. Theta modulated cells were defined as those whose signal to noise ratio (S2N: ratio of average power in 2 Hz band centred on the theta peak to average power in the rest of the 3–25 Hz range) was greater than that seen in 99/100 shuffles where spike times were randomly set. Only a small number of grid cells were significantly theta modulated (27 out of 227 baseline and probe grid cell trials [11.95%], 11.86% of baseline trials, 10.34% of CNO probe trials, 14.00% of saline probe trials, Supp Fig. 5B). The small number of grid cell trials with significant theta modulation precluded full analysis or hypothesis testing of the effect of CNO on the temporal aspects of their firing, and we therefore instead focused on a larger population of theta modulated non-spatial cells (125 out of 145 baseline and probe trials with significant theta modulation [86.21%], Supp Fig. 5A). As in the LFP, after injection of CNO, the theta burst frequency of these cells was reduced, without change in the strength of theta modulation (Fig. 2A–C; RM-ANOVA, trial*drug interaction; frequency: *F*_*(1*,6*)*_ = 30.934, *p* = 0.0014; S2N: *F*_*(1,6)*_ = 0.20, *p* = 0.67). The relationship between theta burst frequency and running speed was also assessed. To do so, power spectra generated from the temporal autocorrelograms of spike trains seen in 2 second windows across the trial were averaged together into 4 cm/s speed bins, with the theta frequency of maximum power identified in each bin. Administration of CNO shifted the speed-frequency relationship downwards (Fig. 2D). Again, this change corresponded to a reduction in parameter A of the exponential fit, whereas no change was seen in parameters B and C (Fig. 2E–G; RM-ANOVA, trial*drug interaction; parameter A: *F*_*(1,3)*_ = 40.40, *p* = 0.008, parameter B: *F*_*(1,3)*_ = 3.66, *p* = 0.15; parameter C: *F*_*(1,3)*_ = 5.97, *p* = 0.092). The same conclusions were reached when averaging at the level of cell rather than animal, to increase the number of data points (Supp Fig. 5C–G). Hence, as in the LFP, modulation of septal cholinergic activity reduced the burst frequency of theta modulated neurones by shifting the theta frequency vs running speed relationship to lower frequencies, without affecting the depth of theta frequency modulation across different speeds.

**Figure 3:**
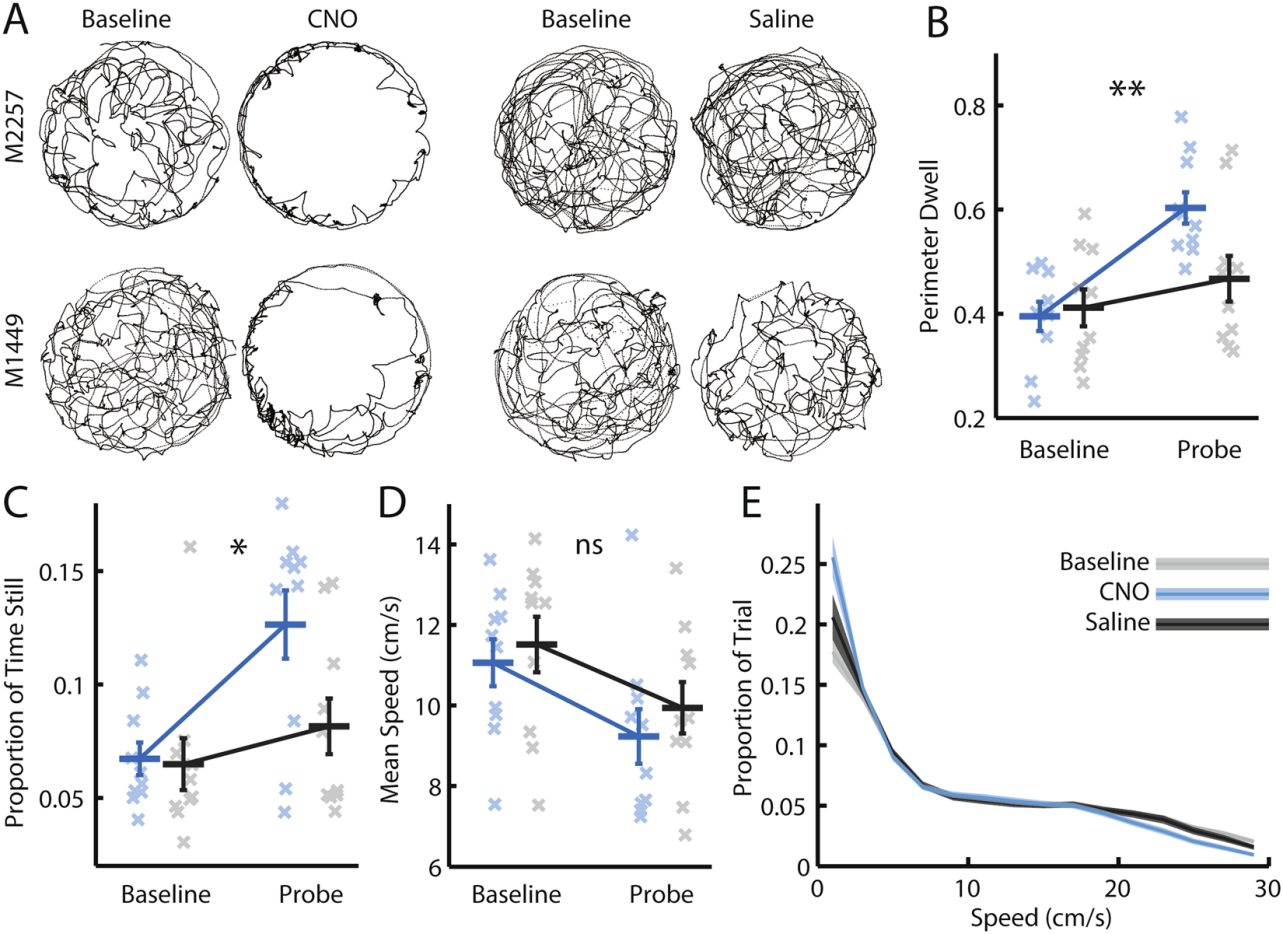
Modulating medial septal cholinergic activity results in a pattern of behaviour normally seen during exposure to novel environments. (**A**) Cumulative position plots from two animals (one in each row), indicating coverage of the environment in the first five minutes of baseline, CNO, and saline probe trials. (**B**–**D**) Blue = CNO, black = saline. Bars indicate mean ± SEM across all animals in each group. Crosses indicate average value for each animal in each group. Stars indicate RM-ANOVA baseline vs probe * CNO vs saline interaction significance. ***p < 0.001, **p < 0.01, *p < 0.05, ns = p > 0.05. (**B**) Proportion of the first five minutes of the trial spent within 10 cm of the walls of the enclosure (‘perimeter dwell’). (**C**) Proportion of the first five minutes of the trial spent sitting still (running speed < 1 cm/s). (**D**) Mean running speed across the whole trial. (**E**) Mean ± SEM proportion of the whole trial spent at each 2 cm/s speed bin across all baseline, CNO, and saline probe trials respectively.

At the start of trials following injection of CNO, mice displayed a pattern of behaviour normally seen during exposure to novel environments. In the first five minutes of CNO probe but not saline probe trials, mice exhibited a clear tendency to run predominantly around the perimeter of the environment (Fig. 3A). Indeed, injection of CNO led to a significant increase in the proportion of the first five minutes of the trial that mice spent within 10 cm of the walls of the enclosure (‘perimeter dwell’, Fig. 3B; RM-ANOVA, trial*drug interaction; *F*_*(1,9)*_ = 17.103, *p* = 0.0025), and not moving (Fig. 3C; RM-ANOVA, trial*drug interaction; *F*_*(1,9)*_ = 7.251, *p* = 0.025). These changes were seen despite there being no effect of CNO on the average running speed across the trial (Fig. 3D; RM-ANOVA, trial*drug interaction, *F*_*(1,9)*_ = 0.11, *p* = 0.75). There was also no difference in the average distribution of running speeds across CNO and saline probe trials (Fig. 3E; Kolmogorov-Smirnov test, *p* = 0.998). Therefore, in addition to modulating theta frequency, alteration of the excitability of MS cholinergic neurones also led mice to spend a greater proportion of the beginning of subsequent trials near the perimeter of the enclosure and not moving.

Three control animals transfected with the fluorophore virus AAV2-hSyn-DIO-mCherry were exposed through intraperitoneal injections to 1, 3, and 5 mg/kg doses of CNO, and equivalent volumes of saline. In contrast to mice injected with the hM3Dq virus, no effect of CNO was observed on peak LFP theta frequency (Supp Fig. 3A), LFP theta frequency vs running speed exponential fit parameter A (Supp Fig. 3B), perimeter dwell (Supp Fig. 3C), or the proportion of time spent sitting still (Supp Fig. 3D). Mixed model ANOVA (with hM3Dq vs DIO-mCherry control animals as a between-subject factor, and baseline vs CNO probe vs saline probe as a within-subject factor) were used to assess whether the change in these measures between drug conditions differed between hM3Dq and control mice. Indeed, significant interaction terms indicated a clear difference between hM3Dq and control mice in the change in exponential fit parameter A and perimeter dwell values across drug conditions (Supp Fig. 3J,K; MM-ANOVA mouse-type*drug interaction; parameter A: *F*_*(2,10)*_ = 12.37, *p* = 0.0019; perimeter dwell: *F*_*(2,10)*_ = 5.90, *p* = 0.020). The interaction effects for peak theta frequency and time spent still were not significant however (Supp Fig. 3I–L; MM-ANOVA mouse-type*drug interaction peak theta frequency: *F*_*(2,10)*_ = 2.17, *p* = 0.165; time still: *F*_*(2,10)*_ = 1.16, *p* = 0.35), probably due to the relatively low power of the statistical test (experiments were only performed on three control mice) and the relatively poorer and more variable behaviour of the control mice: in contrast to the hM3Dq mice, the controls were food restricted for a shorter period and less thoroughly behaviourally trained, as extensive coverage of the enclosure was not required given the absence of grid cell recordings, potentially affecting measures sensitive to the animal’s running behaviour, including peak theta frequency and time sitting still. No dose-dependent effects were observed in the control mice across these same four measures between the 1, 3, and 5 mg/kg concentrations of CNO administered (Supp Fig. 3E–H). The changes observed in animals injected with the hM3Dq virus cannot be explained by the fact that CNO probe trials always followed saline probe trials on ‘dual-drug’ days in which both saline and CNO probe trials were carried out: when only data from days in which only either a CNO or saline probe trial were performed, with the order of days counterbalanced between animals, all significant results were maintained (Supp Fig. 4). These data thus give credence to the conclusion that the effects described here result from modulation of the excitability of MS cholinergic neurones, arising from CNO acting on exogenously expressed hM3Dq receptors, rather than through either CNO or Clozapine acting on endogenous receptors.

**Figure 4:**
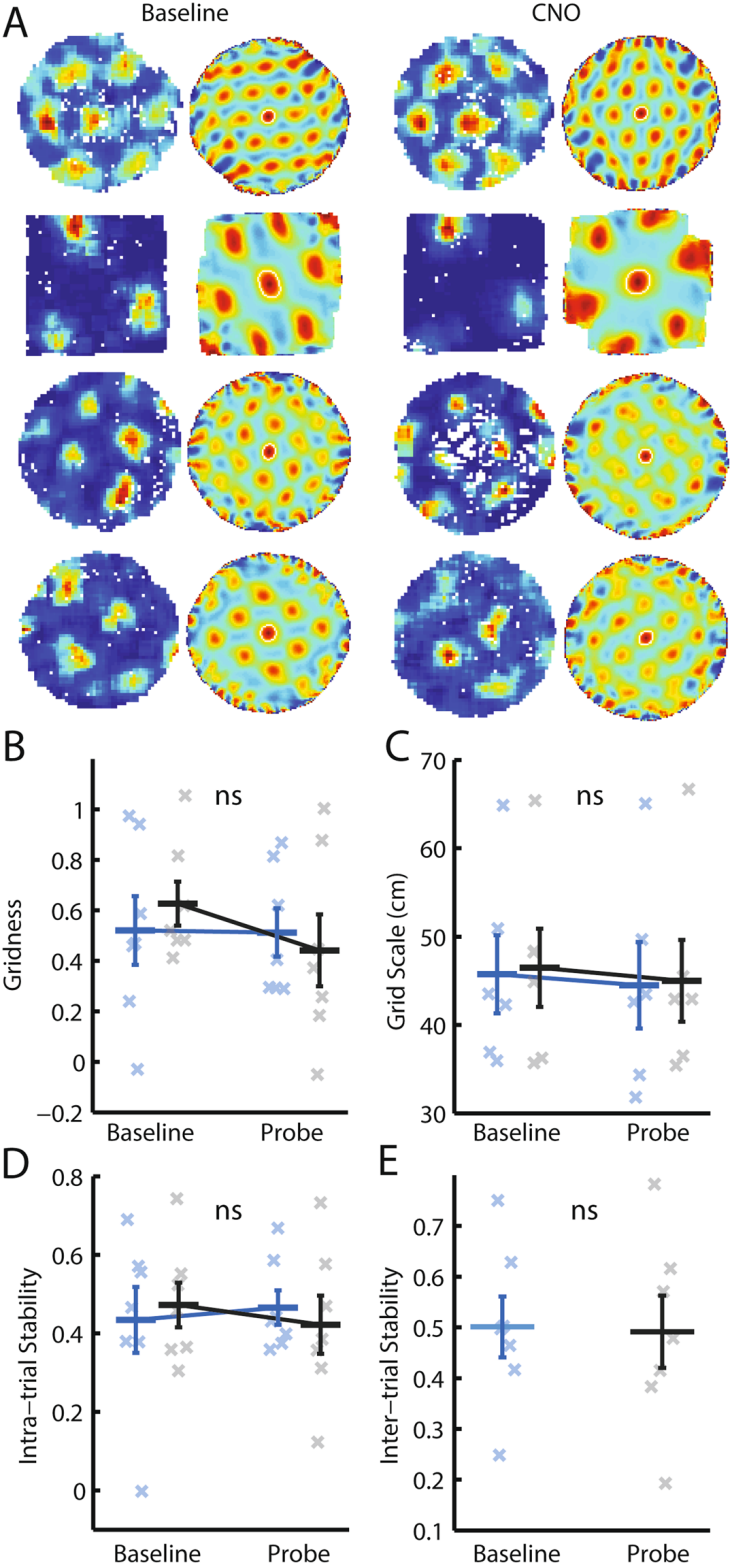
Modulating medial septal cholinergic activity has no effect on grid cell firing patterns. (**A**) Example ratemaps and spatial autocorrelograms from four grid cells (one in each row), in a baseline trial (left hand side) and a CNO probe trial (right hand side). (**B**–**E**) Blue = CNO, black = saline. Bars indicate mean ± SEM across all animals in each group. Crosses indicate average value for each animal in each group. Only cells with gridness > 0.3 in either a baseline or probe trial are included. (**B**–**D**) Stars indicate RM-ANOVA baseline vs probe * CNO vs saline interaction significance. ***p < 0.001, **p < 0.01, *p < 0.05, ns = p > 0.05. (**B**) Degree of hexagonal regularity of grid cell firing patterns, as measured by the gridness score. (**C**) Grid cell scale in cm, measured as the median distance to the six peaks closest to the centre of the autocorrelogram. Only trials with gridness > 0.3 are included. (**D**) Intra-trial stability of grid cell firing patterns, as measured by the value at the centre of a cross-correlogram constructed from two ratemaps, one from each half of the trial. (**E**) Inter-trial stability of grid cell firing patterns, as measured by the value at the centre of a cross-correlogram constructed from two ratemaps, one from the baseline and one from the probe trial. No significant difference was found between the groups using a paired t-test.

Grid cells were recorded in 7 of the 10 mice. A cell was classified as a grid cell if it exhibited hexagonally periodic spatial firing (gridness score > 0.3 in either a baseline or probe trial). Following injection of CNO, little change was observed when comparing ratemaps of the same grid cells between baseline and CNO probe trials (Fig. 4A). Indeed, there was no significant change in gridness scores measured following injection of CNO (Fig. 4B; RM-ANOVA, trial*drug interaction; *F*_*(1,6)*_ = 1.05, *p* = 0.34). Neither was there a significant effect on grid scale, when looking at cells with gridness > 0.3 in both a baseline and probe trial (Fig. 4C; RM-ANOVA, trial*drug interaction; *F*_*(1,5)*_ = 0.021, *p* = 0.89). In analysing whether CNO affected the intra-trial stability of grid-patterns, bin-wise Pearson correlations were calculated between ratemaps corresponding to the first and second half of each trial; no effect of CNO was observed (Fig. 4D; RM-ANOVA, trial*drug interaction; *F*_*(1,6)*_ = 0.75, *p* = 0.42). Similarly, we assessed the inter-trial stability of grid cells using the same procedure to compare baseline ratemaps with saline and CNO probe trials. CNO was not found to affect the spatial stability of grid firing across trials (Fig. 4E; paired t-test, *t*_*6*_ = −0.38, *p* = 0.72). The same conclusions were reached when averaging at the level of cell rather than animal to increase the number of data points (Supp Fig. 6A,B). That is, while altering septal cholinergic activity modulated theta frequency and behaviour, no effect was seen on grid cell firing patterns.

**Figure 5:**
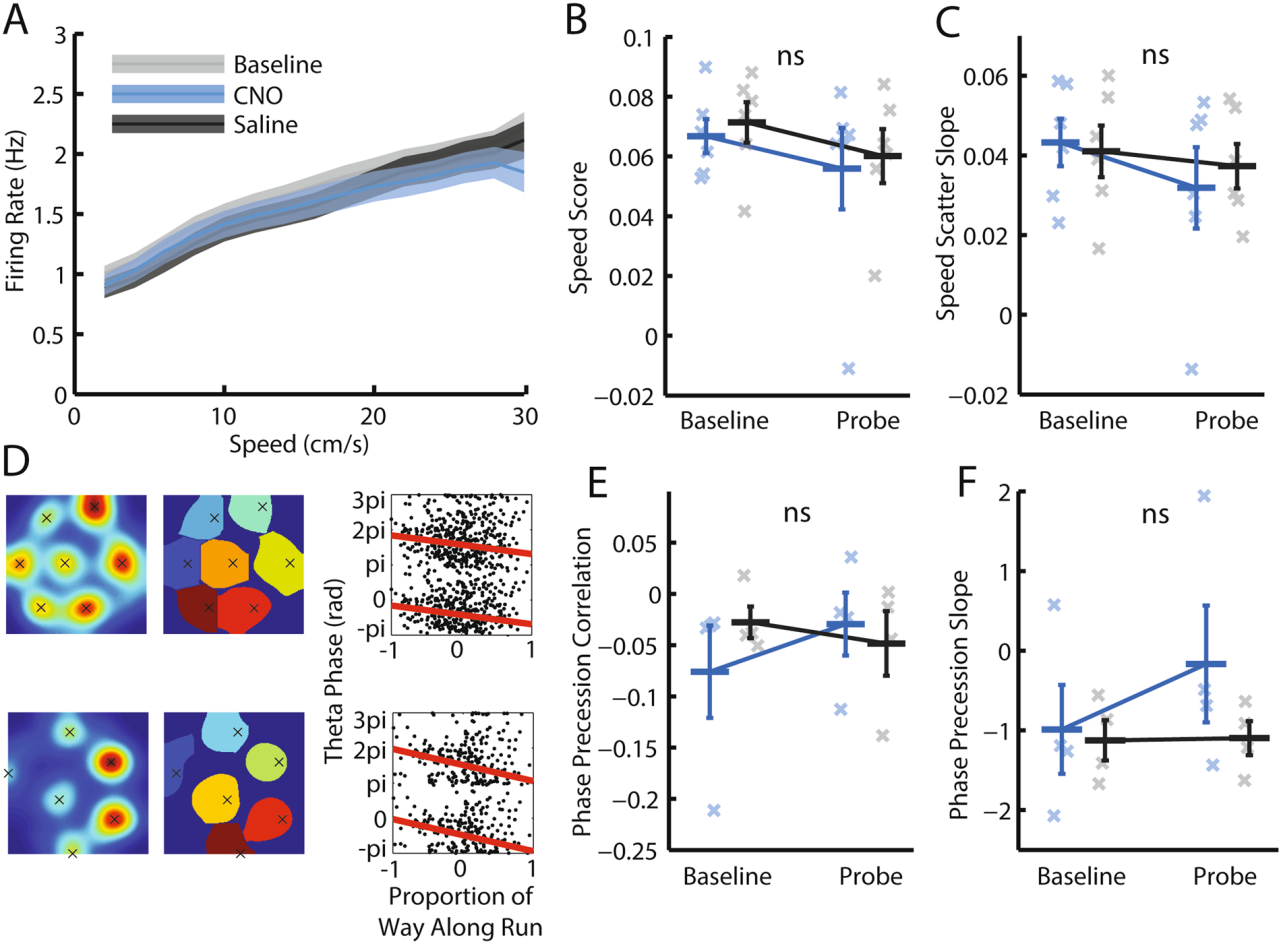
Modulating medial septal cholinergic activity has no effect on putative grid cell speed signals. (**A**) Mean ± SEM firing rate of grid cells as a function of running speed, averaged across all baseline, CNO, and saline probe trials respectively. Only grid cell trials with significant speed modulation (determined by a shuffle procedure) are included. (**B**,**C**,**E**,**F**) Blue = CNO, black = saline. Bars indicate mean ± SEM across all animals in each group. Crosses indicate average value for each animal in each group. Stars indicate RM-ANOVA baseline vs probe * CNO vs saline interaction significance. ***p < 0.001, **p < 0.01, *p < 0.05, ns = p > 0.05. (**B**,**C**) Only grid cells with significant speed modulation in either a baseline or probe trial are included. (**B**) Grid cell speed scores: the Pearson correlation between a cell’s instantaneous firing rate and the animal’s running speed. (**C**) Grid cell speed slope: the slope of the regression line which best fitted the relationship between instantaneous firing rate and running speed. (**D**) Two example CNO probe trials in which significant grid cell phase precession was seen. One cell is plotted in each row. Left-hand side: smoothed firing rate map, with crosses indicating field peaks. Centre: Watershed-isolated valid fields. Right: scatter of theta phase as a function of proportion of distance along run through the field. Red line indicates best fitting circular-linear regression slope. (**E**,**F**) Only grid cells with significant phase precession in either a baseline or probe trial are included. (**E**) Circular-linear correlation values relating theta phase to proportional distance through the field. (**F**) The slope of the circular-linear phase precession regression line.

Given the absence of change in grid cell firing patterns, we asked whether modifying MS cholinergic activity affected putative speed signals seen in grid cells. First, we analysed changes in the firing rates of grid cells seen as a function of running speed. Specifically, following Kropff *et al*. 2015 ^41^, we calculated the instantaneous firing rate of each grid cell, using linear regression to identify the slope and correlation (‘speed score’) of its relationship with running speed. A grid cell was identified as significantly speed modulated if, in either a baseline or probe trial, it had both gridness > 0.3 and a speed score exceeding 99% of a null distribution of 1000 speed scores generated by randomly permuting its spike train relative to the animal’s running speed. No effect of CNO could be seen in the binned average firing rate vs running speed plots of speed modulated grid cells (Fig. 5A). Indeed, in animals with speed modulated grid cells, there was no effect of CNO on the speed score, nor the slope of the firing rate vs running speed regression line (Fig. 5B,C; RM-ANOVA, trial*drug interaction; speed score: *F*_*(1,5)*_ = 0.00079, *p* = 0.98; slope: *F*_*(1,5)*_ = 0.41, *p* = 0.55). An animal’s running speed is also thought to be encoded in the difference in frequency of multiple theta oscillations whose frequency vs speed relationship have differing slopes^18^. This encoding mechanism is thought to manifest as theta phase precession, the tendency of grid cells to fire at earlier phases of the theta cycle as the animal moves through grid fields^42^, and we therefore asked whether theta phase precession was affected by administration of CNO. Following CNO injection, theta phase precession could still be observed in grid cells (Fig. 5D), and there was no effect on the correlation or slope of grid cells assessed as significantly phase precessing by a shuffle procedure (Fig. 5E,F; RM-ANOVA, trial*drug interaction; *F*_*(1,3)*_ = 1.10, *p* = 0.38; *F*_*(1,3)*_ = 1.51, *p* = 0.31). Repeating the speed modulation and phase precession analyses averaging at the level of cell (rather than animal) to increase the number of data points did not change these conclusions (Supp Fig. 6C–F). The absence of change in grid cell firing patterns was therefore consistent with a lack of change in putative speed signals in grid cells following modulation of MS cholinergic tone.

## Discussion

Aiming to illuminate the contribution of cholinergic neurones to the septum’s theta rhythmogenesis, its encoding of running speed, and its support of grid cell firing, we modulated the activity of MS cholinergic neurones while recording local field potentials and from single units in the medial entorhinal cortex of freely foraging mice. Using DREADDs to modulate the excitability of septal cholinergic cells reduced theta frequency by shifting its saturating-exponential relationship with running speed to lower frequencies, and led mice to exhibit behaviours at the start of trials normally seen on exposure to novel environments. Despite these changes, no effect was seen on grid cell firing patterns, consistent with a lack of change in either the theta-frequency or firing-rate speed signals potentially used by grid cells.

A reduction in theta frequency following modulation of cholinergic activity is consistent with evidence demonstrating that application of the muscarinic receptor agonist carbachol reduces the frequency of subthreshold membrane potential oscillations^43^, and resonance^44^, of medial entorhinal layer-II stellate cells *in-vitro*. Through application of the acetylcholinesterase-inhibitor physostigmine, similar results have also been observed *in-vivo* ^45^. Blockade of muscarinic receptors with systemic administration of scopolamine has also been observed to reduce theta frequency in behaving animals, though in contrast to the present results, through a flattening of the theta frequency vs running speed slope^24^. Further, lesions specifically targeting MS cholinergic neurones have been found to reduce the power of theta oscillations without affecting their frequency^21,22^. The current results therefore complement a growing body of evidence demonstrating a role for MS cholinergic neurones in modulating movement-related theta oscillations, though one in which their precise functional contribution remains unclear. Some variability in the described results likely stems from differences in the techniques employed both to modulate cholinergic tone and record theta. For example, recording in the hippocampus rather than the entorhinal cortex may lead to observation of a change in power without a change in frequency of theta oscillations, because of an absence of the stellate cells whose subthreshold oscillatory dynamics are modulated by acetylcholine^44^. Similarly, non-linear responses to acetylcholine concentration, as well as adaptation over time, may mean that the impact of any intervention depends on whether it increases or decreases cholinergic tone, is acute or chronic. Further experiments are also required to identify whether the observed reduction in theta frequency is due to local connections of cholinergic neurones within the MS, or direct projections to the entorhinal cortex.

The strongly non-linear relationship between theta frequency and running speed observed here is unlikely to be an artefact of the analysis, as the same procedure was capable of correctly identifying linear and sinusoidal relationships in artificially-generated data. Further, though not remarked upon, a number of observations of non-linear increases in theta frequency have been made in the past^24,39,46^; though, to the best of our knowledge, this is the first explicit quantification of the non-linearity. The saturating-exponential relationship consistently observed here means that the fastest rate of change in theta frequency is seen at the lowest running speeds. It is possible that this rapid change in frequency represents a transition between immobility-related Type II, and movement-related Type I theta. However, in the current data, analysis using narrow-width (0.25 cm/s) speed bins consistently indicated a smooth increase in frequency, rather than a discontinuous jump between low- and high-frequency theta states. It is worth nothing that oscillatory interference models require that the differences in the frequencies of the constituent oscillators vary linearly with running speed, but not necessarily that the LFP speed-frequency relationships themselves be linear^47^.

Despite the observed effects on theta frequency, no change was seen in the firing patterns of recorded grid cells. The consistency of grid firing patterns was concordant with an absence of change in both the theta-frequency and firing-rate running speed signals thought used to update grid firing. The lack of change in these running speed codes suggests that such activity does not acutely depend on septal cholinergic neurones. Given the profound disruption of these signals during inactivation of the MS^16,17,32^, the present data instead imply that such functions depend on GABAergic or glutamatergic septal circuits. The absence of change in these signals prevent conclusions being drawn regarding the alternative hypotheses of oscillatory interference and continuous attractor models as to which of these two speed codes is central to the updating of the grid firing. In the present experiment, modulating septal cholinergic activity had no effect on the depth of modulation of theta frequency seen as a function of running speed, in contrast to the flatter profile seen following systemic administration of the muscarinic receptor antagonist scopolamine^48^. This difference may explain why little change was seen here in grid cell firing patterns, in contrast to the breakdown of periodic firing previously reported^48^. The cause of the contrasting effects of acetylcholine manipulations on the theta frequency slope between the present and previous studies remains to be seen, but may relate to scopolamine acting as a global antagonist of cholinergic function, in contrast to the targeted modulation of septal cholinergic neurones used here. Alternatively, as noted, without direct recordings of cholinergic neurones in the present study, it remains equivocal what effect the DREADD manipulation ultimately had on cholinergic firing, and this inconsistency may result from differences in the net effect and time course the alternative interventions have on septal cholinergic tone.

The tendency of rodents to remain still and avoid the centre of a novel open enclosure has long been used as a test to identify anxiolytic effects of drugs^49^, while electric shocks have been shown to increase rats’ tendency to ‘freeze’, and remain near the perimeter of novel environments^50^. As such, these freezing and ‘thigmotactic’ behaviours are closely associated with both novelty and anxiety. In the current experiment, the increased proportion of time spent in the periphery of the enclosure and not-moving cannot be accounted for by an anxiogenic effect of the intraperitoneal injection alone, as the increase in both measures seen following injection of CNO was significantly greater than that seen following injection of saline. Interestingly, the reduction in theta frequency here is similar to that observed following injection of anxiolytic drugs in rats^51^, while the behavioural changes are redolent of anxiogenesis or novelty. Acetylcholine is closely related to learning and memory^52,53^, and observed increases in cholinergic tone in novel contexts^54^ are thought to drive the encoding of new memories in the hippocampal formation^52^. The novelty-like behaviours seen here after modulating the activity of MS cholinergic neurones could therefore be interpreted as the result of inducing a sense of novelty in the mice, though the current data cannot distinguish this interpretation from an increase in anxiety.

The Cre-dependent virus used here meant that the DREADD hM3Dq was expressed exclusively in cholinergic neurones of the MS. hM3Dq has been widely used as a neuroscientific tool, as injection of its agonist CNO leads to an increase in the excitability of hM3Dq expressing neurones^35^ (though likely only after conversion to Clozapine^36^). Indeed, activation of hM3Dq receptors by CNO can lead neurones to emit bursts of action potentials^35^. However, the effect of this manipulation on the activity patterns of the slow-firing cholinergic neurones of the MS^55^ remains to be seen. Further, given that septal cholinergic neurones are interconnected with other neural subgroups of the MS, including GABAergic neurones^37,56^, the ultimate effect on the activity of both cholinergic and non-cholinergic components of the MS circuitry of the hM3Dq manipulation in the present experiment is unknown. Indeed, in certain neurones, using genetic tools to elicit extended excitation has been shown to lead to reduced firing, for example through depolarising block^57^. As such, while the robust effects of this manipulation on both theta oscillations and the mice’s behaviour can be inferred to arise from a perturbation of the MS network that originates with the modulation of the excitability of cholinergic neurones, without direct recordings from septal neurones, the precise nature of the intervention remains uncertain. While beyond the scope of the current experiment, fibre-photometry or optogenetic-tagging could be used to record from specific neural subgroups of septal neurones, such that the effect of CNO administration on each genetic subtype in the MS could be identified.

In sum, the data presented here provide evidence for a role of MS cholinergic neurones in modulating movement-related theta oscillations, particularly in determining their frequency. Further, an increase in time sitting still and an avoidance of the centre of the environment suggests that the DREADD manipulation of cholinergic neurones gave rise to a sense of novelty or anxiety. In comparison to the profound effect of MS inactivation on theta oscillations^5,6^, firing-rate speed signals^32^, and grid cell firing^16,17^, the preservation of these neural processes in the present data suggest they do not acutely depend on septal cholinergic neurones, and thus that they may instead arise principally from the activity of GABAergic or glutamatergic cells in the MS. However, to confirm whether the present data’s suggestion of this hypothesis is valid, one would ideally selectively and bidirectionally modulate the activity of septal cholinergic neurones. In this experiment, a lack of direct recordings however means that the net effect of the DREADD manipulation on the activity of both cholinergic and non-cholinergic neural subgroups of the MS remains uncertain.

## Methods

A full account of the methods and analyses employed is provided in the Supplementary Information section.

All work was conducted within the terms of appropriate Project and Personal Licenses approved by University College London and the UK Home Office. 13 ChAT-IRES-Cre mice were injected in the medial septum (0.7–0.9 mm anterior to bregma, 0.8mm lateral to bregma, 12° angle towards midline, 4 × 300 nl injections at 4.3, 3.9, 3.5 and 3.1 mm ventral from brain surface) with the Cre-dependent virus AAV2-hSyn-DIO-hM3D(Gq)-mCherry or the control virus AAV2-hSyn-DIO-mCherry. Two microdrives, each containing four tetrodes, were implanted bilaterally in the medial entorhinal cortex (3.2 mm lateral to lambda, 0.3–0.5 mm anterior to the anterior edge of the transverse sinus, 6° angle in the posterior direction, 0.8–1 mm below brain surface) in the same animals.

At least three weeks after surgery, single-unit and local field potential recordings were made while mice explored familiar 90 × 90 cm square or 1 m diameter circular environments. In seven of the 10 hM3Dq mice, screening was continued until grid cells were recorded. In three of the hM3Dq and the three control-mCherry mice, no grid cells were recorded, and the experiment was commenced while large amplitude theta oscillations could be observed in the LFP. On ‘single drug’ experimental days, following a baseline trial, mice were injected intraperitoneally with 3 mg/kg of Clozapine-N-Oxide(CNO) or an equivalent volume of saline. That is, on single drug days, *either* CNO *or* saline was injected, with the order of the drugs across days counterbalanced between animals. One hour after the injection, a second trial was recorded, termed a ‘CNO probe’ or ‘saline probe’ trial. In addition, four mice also had recordings made under a ‘dual drug’ protocol. Here, following the baseline trial, the mouse was first injected with saline, and after a one hour break, a saline probe trial was recorded. Subsequently, the mouse was then injected with CNO, and after a second one hour break, a CNO probe trial was recorded. Dual drug days thus included three trials, with the saline probe trial always leading the CNO probe trial due to the long-lasting time course of CNO action. The experimental protocol is pictorially represented in Supplementary Fig. 1.

To assess whether there was a significant change in the LFP, the theta bursting of single units, the mice’s behaviour, or the firing patterns of grid cells, appropriate analyses described below were conducted to generate measures which were tested using Repeated Measures ANOVA (RM-ANOVA) at the level of animal. Concretely, for each measure we averaged together values from all qualifying cells and trials to generate four values for each animal: a CNO baseline, a saline baseline, a CNO probe, and a saline probe value. CNO and saline baselines were distinguished by whether they were followed by injection of either CNO or saline. The interaction term of the RM-ANOVA test was then used to assess the null hypothesis that there was no difference between CNO and saline groups in the change in each measure between baseline and probe trials. Further, Mixed Model ANOVA (MM-ANOVA) were used to assess whether the change in these measures between drug conditions differed between hM3Dq-injected experimental mice and DIO-mCherry injected control mice.

Changes in the frequency and power of LFP theta oscillations were assessed using power spectra generated using Welch’s method. Changes in the shape of the theta frequency vs running speed relationship were analysed through evaluation of the parameters in the three-term saturating exponential function *y* = *A* − *B* * e^(−*C* * *x*)^ which best fit the theta frequency vs running speed distribution produced by binning a spectrogram created with Morlet wavelets into speed bins of 0.25 cm/s from 0–30 cm/s. Equivalent analyses of the theta bursting of single units were performed using stationary or windowed power spectra derived from the temporal autocorrelogram of each unit’s spike train. Changes in the mice’s behaviour were assessed through calculation of the time spent within 10 cm of the borders of the enclosure, the time spent sitting still (moving < 1 cm/s), and the average running speed. Changes in the firing patterns of grid cells were assessed through calculation of gridness and scale values from the spatial autocorrelogram of each unit’s firing rate map. Each grid cell’s inter and intra trial stability were assessed using spatial cross-correlograms between the baseline and probe trial, or the first and second half of the each trial respectively. The speed modulation and phase precession of grid cells were assessed following Kropff *et al*.^41^ and Jeewajee *et al*. ^42^ respectively.

Following completion of the experiments, immunohistochemical analysis was performed on coronal brain slices to assess expression of ChAT and mCherry. Slices were imaged to determine the extent of viral expression, and manual cell counting was performed to measure the overlap between neurones expressing ChAT and mCherry across the entire septum.

### Data and code sharing

The authors will make the data and MATLAB analysis code used available upon reasonable request.

## Electronic supplementary material

Supplementary Information
