# Supplementary material for: Modulating medial septal cholinergic activity reduces medial entorhinal theta frequency without affecting speed or grid coding
Source: Sci Rep. 2017 Nov 6;7:14573. doi: 10.1038/s41598-017-15100-6 (PMC5673944; doi:10.1038/s41598-017-15100-6)
Supplement: Supplementary file 1 — Supplementary Information [file 41598_2017_15100_MOESM1_ESM.pdf]

Modulating medial septal cholinergic activity reduces medial entorhinal theta frequency without affecting speed or grid coding

Francis Carpenter, Neil Burgess, Caswell Barry

Supplementary Information

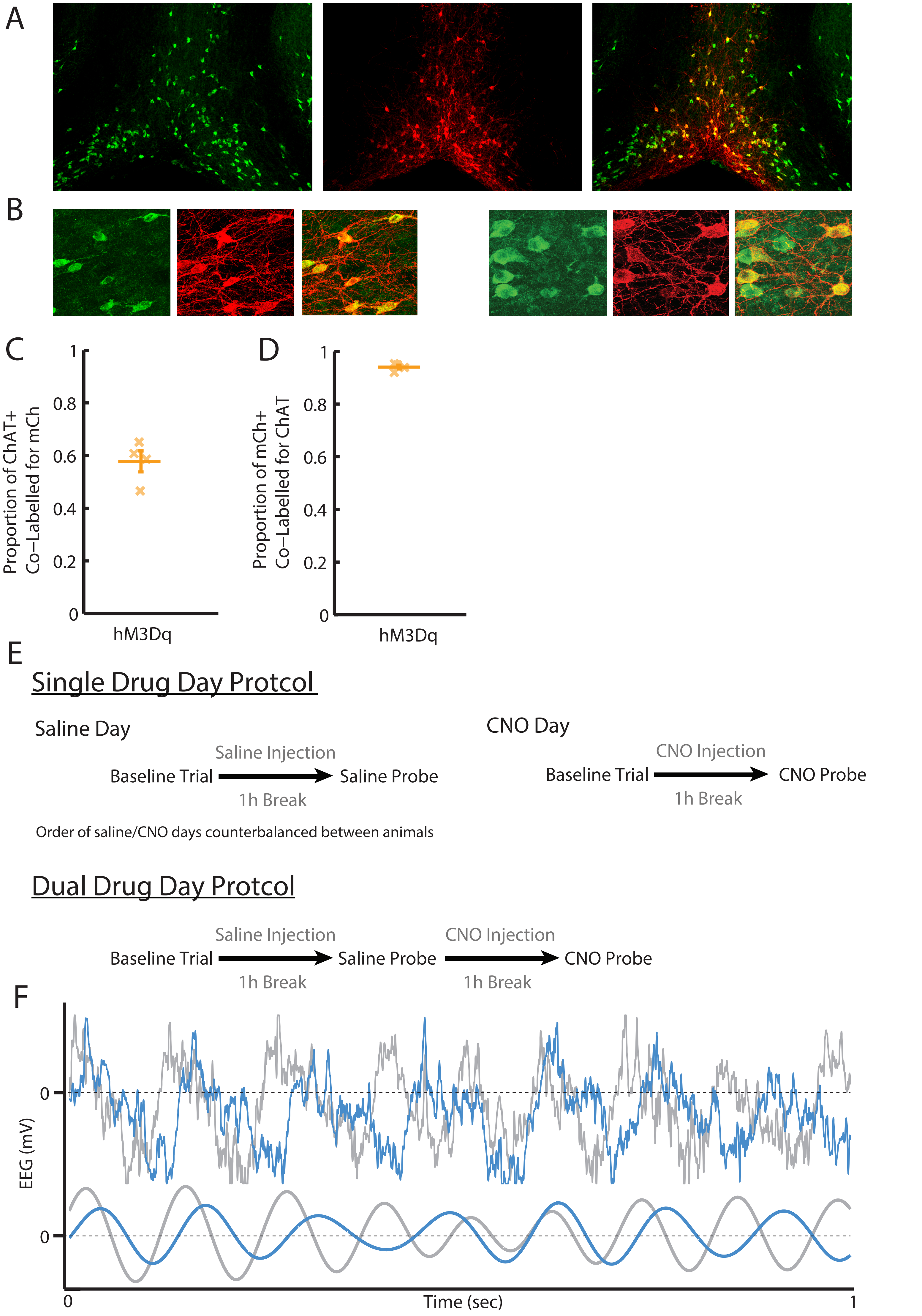
A
B
C
1
0.8
0.6
0.4
0.2
0
Proportion of ChAT+
Co−Labelled for mCh
hM3Dq
D
1
0.8
0.6
0.4
0.2
0
Proportion of mCh+
Co−Labelled for ChAT
hM3Dq
E
Single Drug Day Protcol
Saline Day
Saline Injection
Baseline Trial
Saline Probe
1h Break
CNO Day
CNO Injection
Baseline Trial
CNO Probe
1h Break
Order of saline/CNO days counterbalanced between animals
Dual Drug Day Protcol
Saline Injection
CNO Injection
Baseline Trial
Saline Probe
CNO Probe
1h Break
1h Break
F
0
0
EEG (mV)
0
Time (sec)
1

Supp Fig 1. Confirmation hM3Dq is expressed almost exclusively in medial septal cholinergic neurones; pictorial representation of the experimental protocol; example LFP traces before and after CNO administration. **A**, Example 10x epifluorescence image of coronal brain sections showing ChAT expression (green) and mCherry expression (red) following injection of hM3Dq. **B**, Additional example 40x confocal z-stack images demonstrating tight overlap between ChAT and mCherry expression following injection of hM3Dq. **C-D**, Bars indicate mean ± SEM across all animals, crosses indicate average value for each animal. **C**, Proportion of ChAT+ neurones co-labelled for mCherry, as indicated by manual cell counting. **D**, Proportion of mCherry+ neurones co-labelled for ChAT. **E**, Experimental protocol for recordings made on 'single drug' and 'dual drug' days. **F,** One-second example LFP traces of medial entorhinal theta, recorded from a single mouse during a baseline trial (grey) and after administration of CNO (light-blue). The two one-second epochs were speed matched (baseline: mean speed 17.37cm/s, CNO: 16.97cm/s). The top trace is the raw LFP recorded, the lower trace is the same signal filtered between 6 and 10Hz with a Blackman filter. Traces are displayed from the point at which the raw LFP crosses zero.

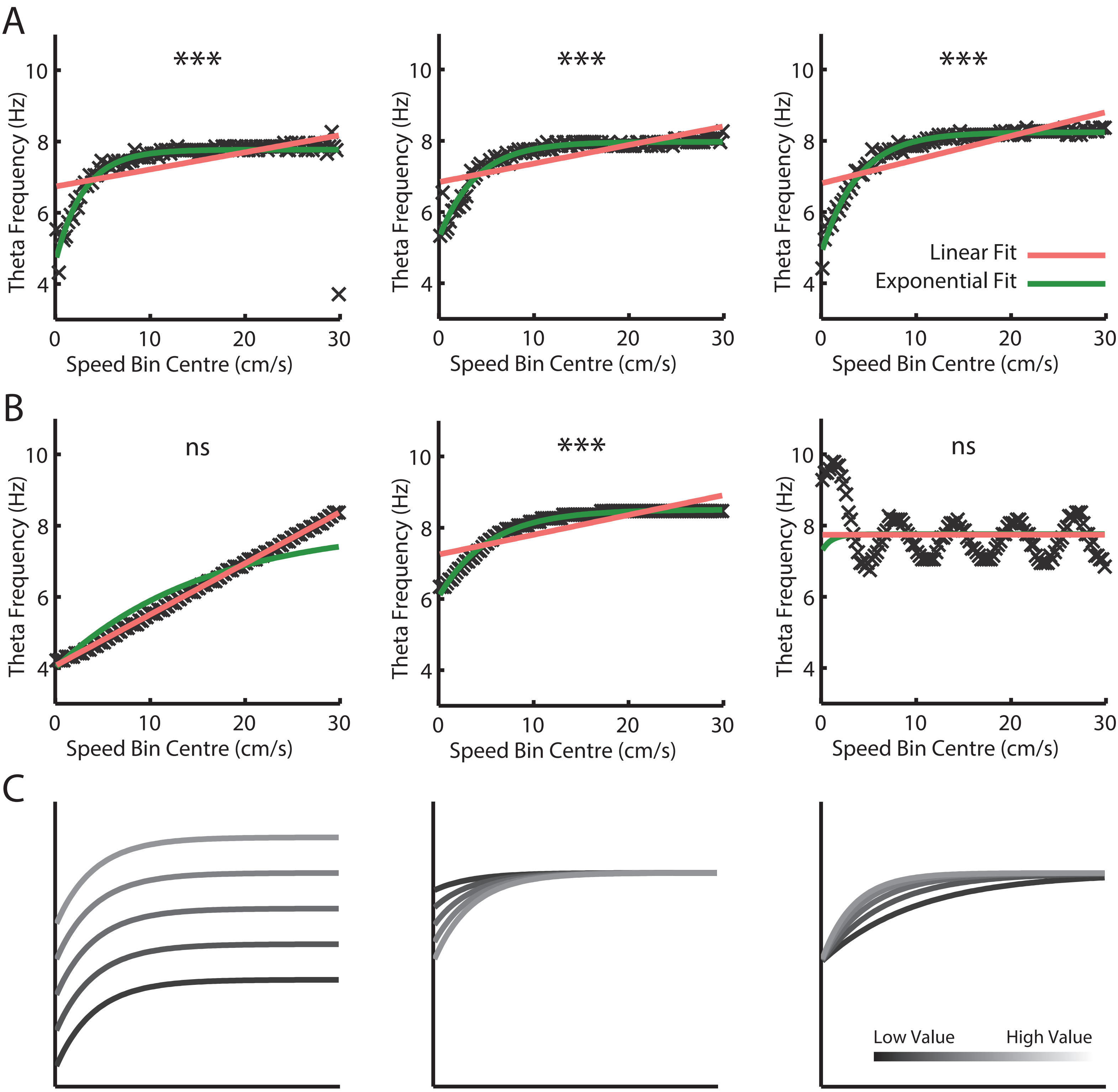
A
B
C
***
ns
Theta Frequency (Hz)
Speed Bin Centre (cm/s)
Linear Fit
Exponential Fit
Low Value
High Value

Supp Fig 2. Examples of observed and artificial LFP theta frequency vs running speed plots demonstrate a clear non-linear relationship between the two. **A-B**, Black crosses indicate theta frequency of maximum power in each running speed bin in the 3-11Hz range. Green line indicates the best three-term exponential fit fitted with non-linear least squares. Red line indicates the best linear fit fitted with linear least squares. Stars indicate whether the exponential model fits the data significantly better than the linear model, accounting for the increased number of free parameters, using the F-test. *** = $p<0.001$, ** = $p<0.01$, * = $p<0.05$, ns = $p>0.05$. **A**, Examples from three baseline trials of observed theta frequency vs running speed profiles **B**, Examples of artificial sinusoidal LFP plots whose frequency was set to vary as a linear (left), saturating-exponential (middle), and sinusoidal (right) function of running speed. **C**, Left to right: plots demonstrating the effect of systematically varying the parameter A, B, and C values respectively in the three-term exponential function used for fitting, while holding the other parameters constant. Darker colours indicate lower parameter values, lighter colours higher values.

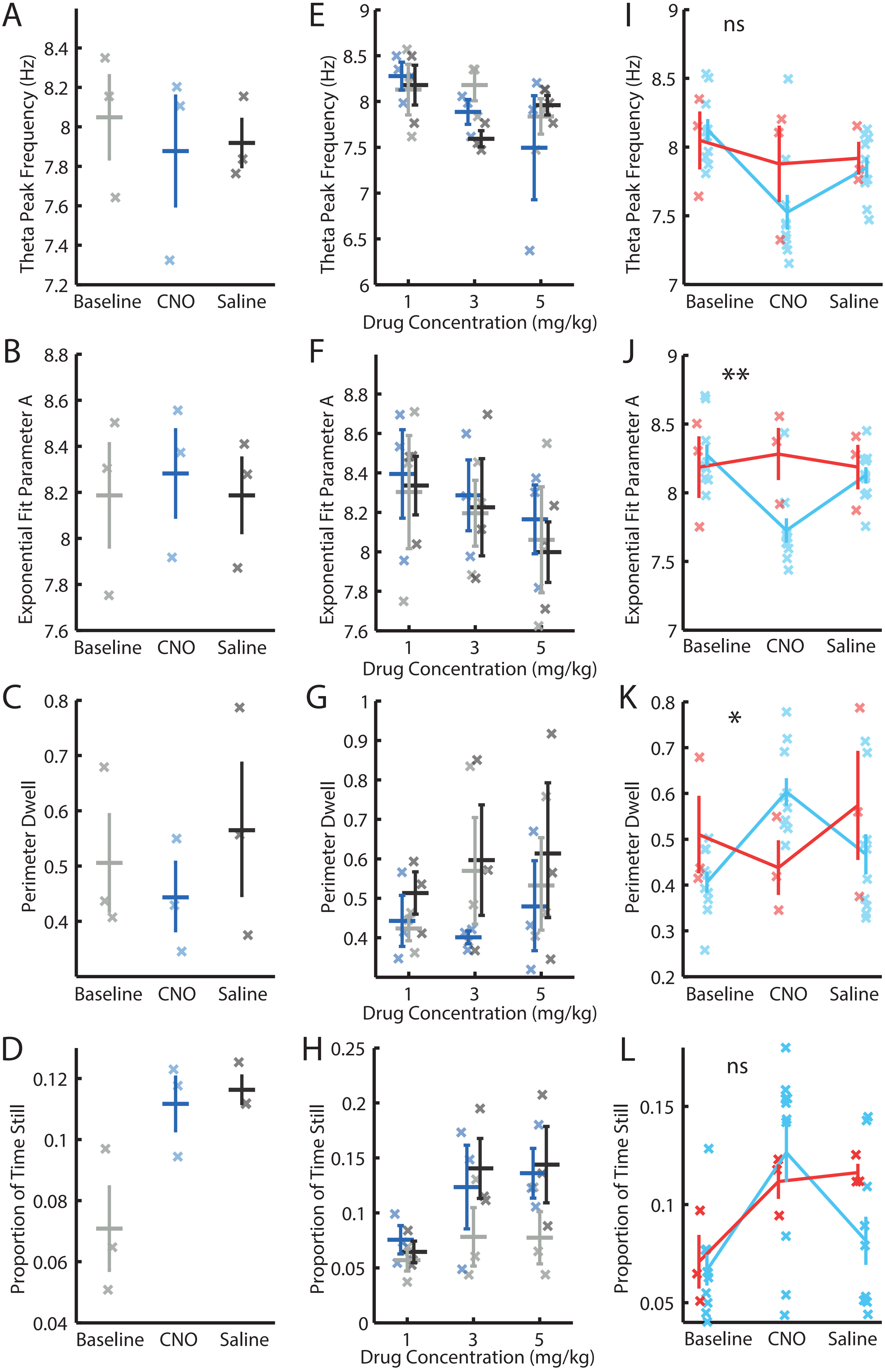

A
B
C
D
E
F
G
H
I
J
K
L
Theta Peak Frequency (Hz)
Exponential Fit Parameter A
Perimeter Dwell
Proportion of Time Still
Baseline
CNO
Saline
Drug Concentration (mg/kg)
ns
**
*
ns

Supp Fig 3. Effects observed following injection of CNO in hM3DQ mice not seen in DIO-mCherry control mice. **A-L,** Bars indicate mean ± SEM across all animals in each group. Crosses indicate average value for each animal in each group**. A-H**, Grey = baseline, blue = CNO, black = saline. **A-D**, Values are averaged across all three drug concentrations administered. **E-H**, Values are separated according to the drug concentration administered. **I-L,** Light blue = hM3Dq mice, red = DIO-mCherry control mice. **A**, **E, I**, Frequency of maximum power in the LFP power spectrum in the theta (6-10Hz) band. **B**, **F, J**, Three-term exponential fit parameter A values that best fitted the LFP theta frequency vs running speed relationship. **C**, **G, K**, Proportion of the first five minutes of the trial spent within 10cm of the walls of the enclosure ('perimeter dwell'). **D**, **H, L**, Proportion of the first five minutes of the trial spent sitting still (speed <1cm/s). Stars indicate MM-ANOVA hM3Dq vs control * drug interaction significance. *** = p<0.001, ** = p<0.01, * = p<0.05, ns = p>0.05.

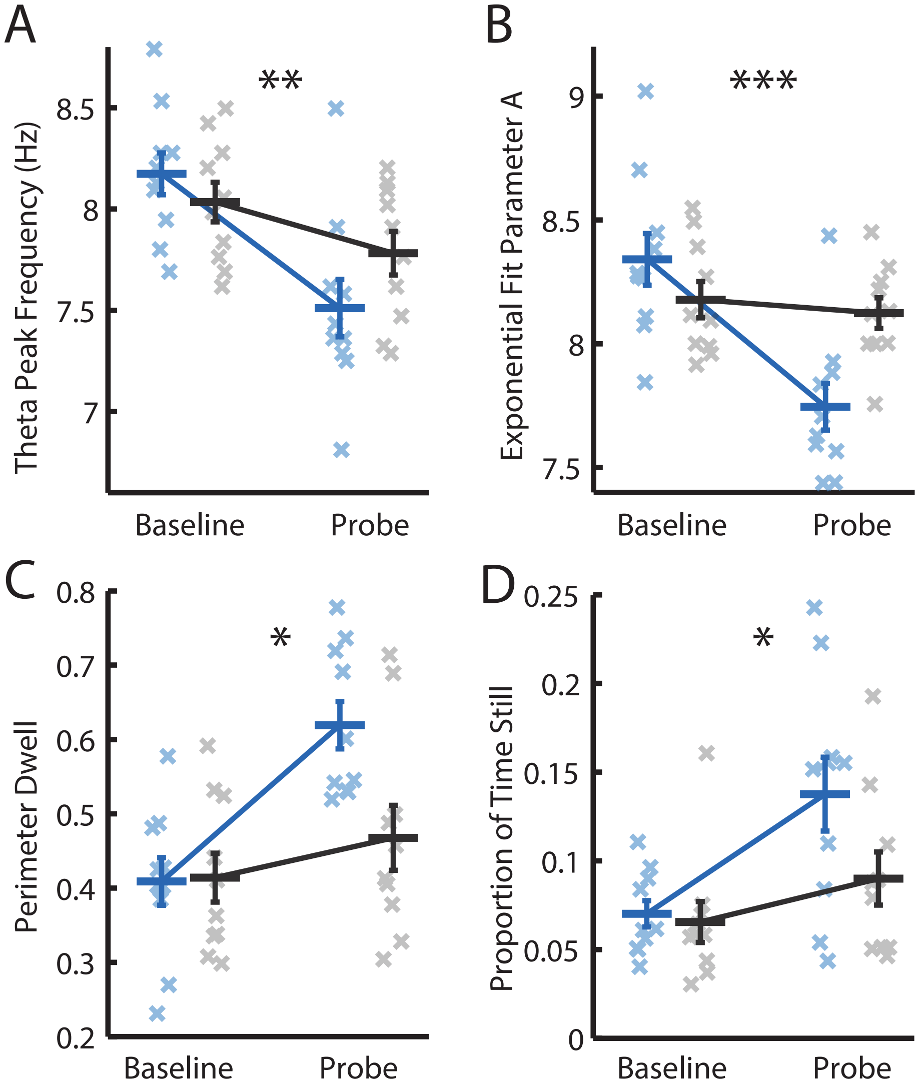
A
B
C
D
Theta Peak Frequency (Hz)
Exponential Fit Parameter A
Perimeter Dwell
Proportion of Time Still
**
***
*
*
Baseline
Probe
8.5
8
7.5
7
9
0.8
0.7
0.6
0.5
0.4
0.3
0.2
0.25
0.15
0.1
0.05
0

Supp Fig 4. Effect of CNO on theta frequency and behaviour still seen when analysis is restricted to 'single drug' days, in which only either CNO or saline were administered. **A-C**, Blue = CNO, black = saline. Bars indicate mean ± SEM across all animals in each group. Crosses indicate average value for each animal in each group. Stars indicate RM-ANOVA baseline vs probe * CNO vs saline interaction significance. *** = $p<0.001$, ** = $p<0.01$, * = $p<0.05$, ns = $p>0.05$. **A**, Frequency of maximum power in the LFP power spectrum in the theta (6-10Hz) band. **B**, Three-term exponential fit parameter A values that best fitted the LFP theta frequency vs running speed relationship. **C**, Proportion of the first five minutes of the trial spent within 10cm of the walls of the enclosure ('perimeter dwell'). **D**, Proportion of the first five minutes of the trial spent sitting still (speed <1cm/s).

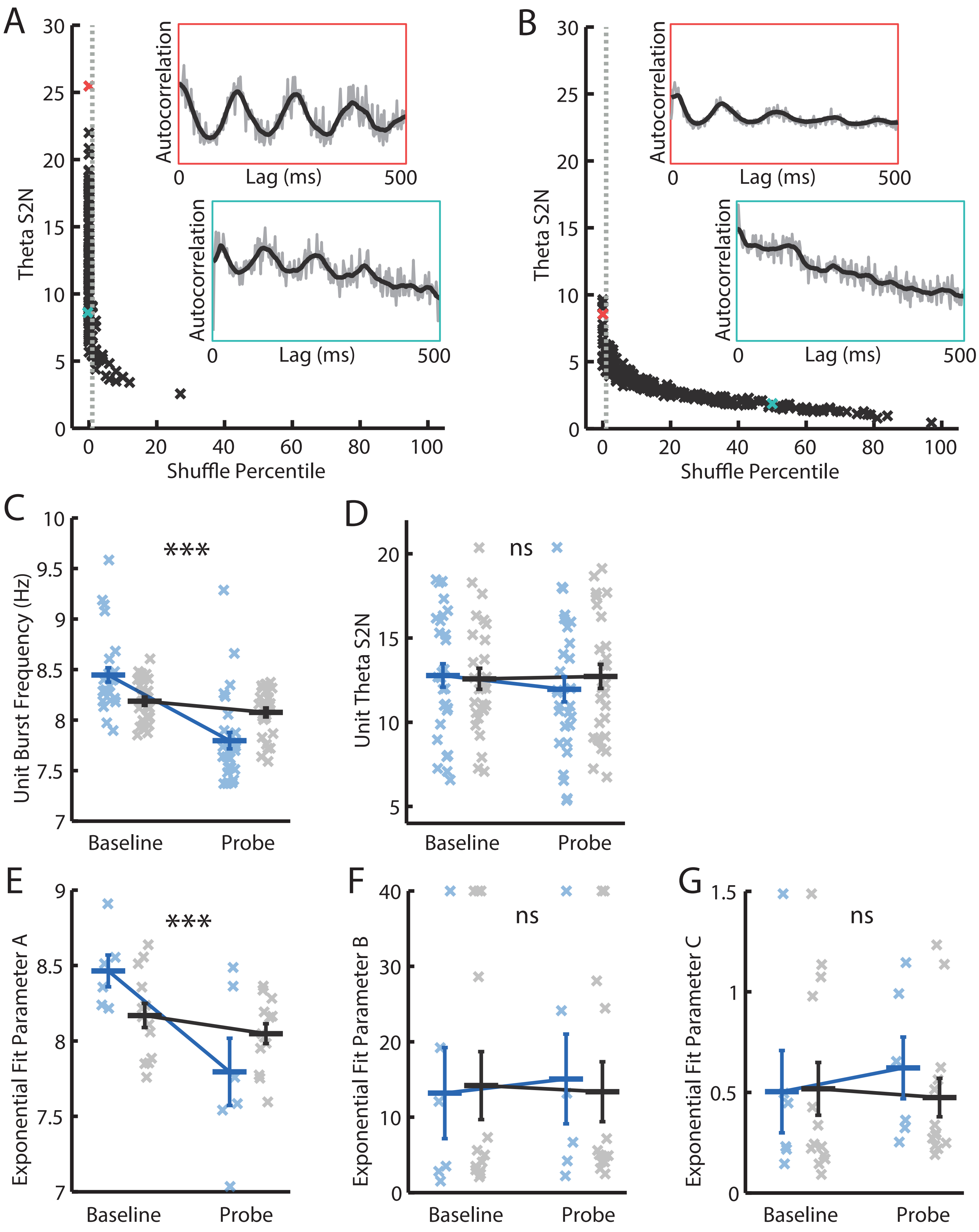
A
B
C
D
E
F
G
Theta S2N
Autocorrelation
Lag (ms)
Shuffle Percentile
***
ns
Unit Burst Frequency (Hz)
Unit Theta S2N
Exponential Fit Parameter A
Exponential Fit Parameter B
Exponential Fit Parameter C
Baseline
Probe

Supp Fig 5. Conclusions of the effect of CNO on theta-modulated unit burst frequency are unchanged by performing analyses averaging at the level of cell **A-B**, Distribution of theta signal to noise (Theta S2N) as a function of location within a distribution of shuffled theta S2N values (shuffle percentile) for theta-modulated non-spatial cells (**A**) and grid cells (**B**). Only baseline and probe trials are included. Vertical dashed grey lines represent the significance threshold. Inset are example temporal autocorrelograms representative of specific S2N values. **C-G**, Blue = CNO, black = saline. Bars indicate mean ± SEM across all cells in each group. Crosses indicate average value for each cell in each group. Only cells with significant theta modulation (assessed by the shuffle procedure) in either a baseline or probe trial are included. Stars indicate mixed-model ANOVA baseline vs probe * CNO vs saline interaction significance. *** = $p<0.001$, ** = $p<0.01$, * = $p<0.05$, ns = $p>0.05$. **C**, Theta burst frequency of single units. **D**, Theta S2N. **E-G**, Only cells with significant theta modulation (assessed by a shuffle procedure) in either a baseline or probe trial and only trials with a significantly better exponential than linear fit (according to the F-test) are included. **E-G**, Parameter A, B, and C values respectively that best fit the single unit theta frequency vs running speed relationship.

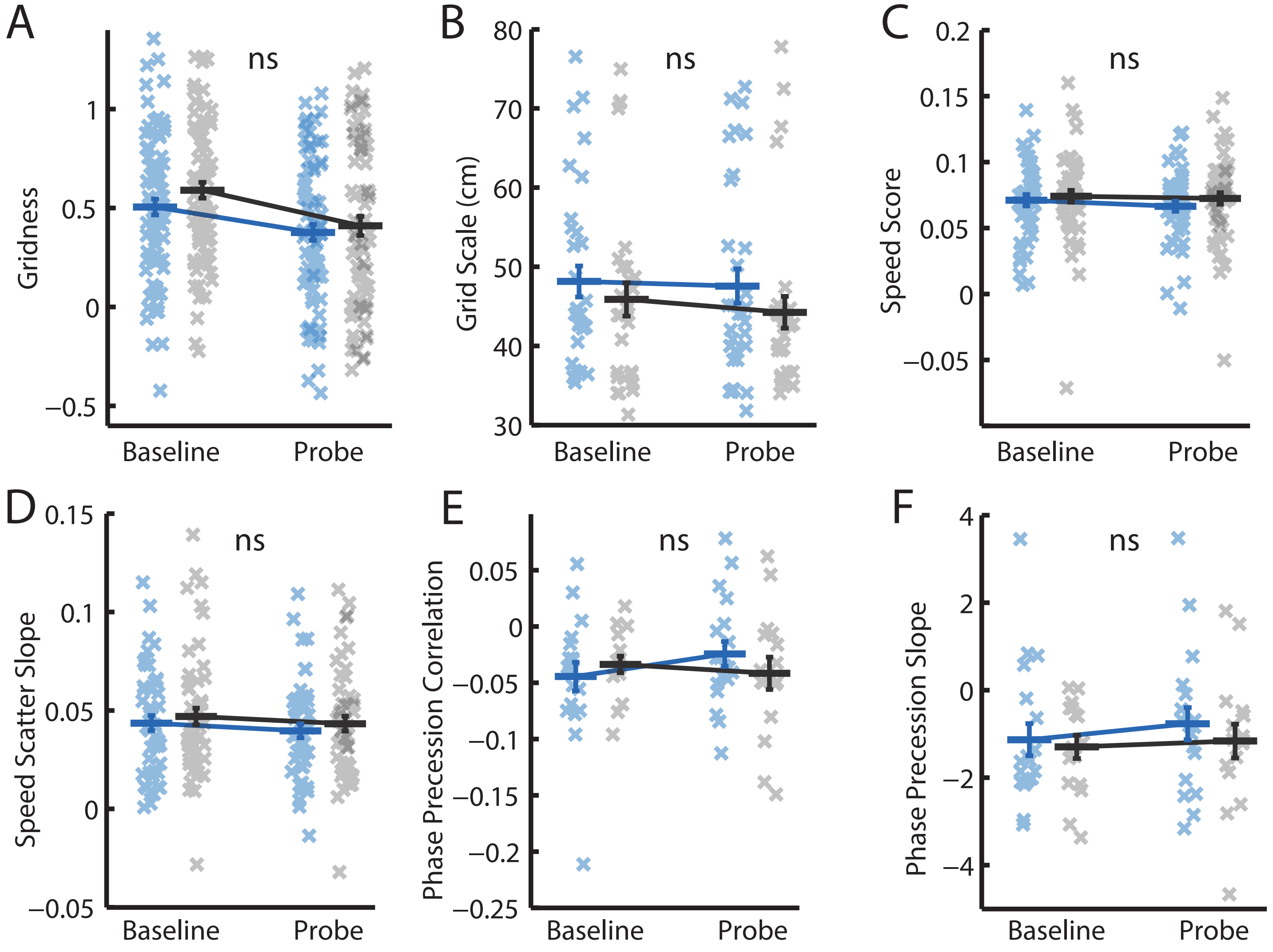

A
Gridness
ns
Baseline
Probe
B
Grid Scale (cm)
ns
Baseline
Probe
C
Speed Score
ns
Baseline
Probe
D
Speed Scatter Slope
ns
Baseline
Probe
E
Phase Precession Correlation
ns
Baseline
Probe
F
Phase Precession Slope
ns
Baseline
Probe

Supp Fig 6. Conclusions of the effect of CNO on grid cell firing patterns are unchanged by repeating analyses averaging at the level of cell. **A-F**, Blue = CNO, black = saline. Bars indicate mean ± SEM across all cells in each group. Crosses indicate average value for each cell in each group. Only cells with gridness > 0.3 in either a baseline or probe trial are included. Stars indicate mixed-model ANOVA baseline vs probe * CNO vs saline interaction significance. *** = $p<0.001$, ** = $p<0.01$, * = $p<0.05$, ns = $p>0.05$. **A**, Degree of hexagonal regularity of grid cell firing patterns as measured by the gridness score. **B**, Grid cell scale in cm, measured as the median distance to the six peaks closest to the centre of the autocorrelogram. Only trials with gridness > 0.3 are included. **C-D**, Only grid cells with significant speed modulation in either a baseline or probe trial are included. **C**, Grid cell speed score: the Pearson correlation between a cell's instantaneous firing rate and the animal's running speed. **D**, Grid cell speed slope: the slope of the regression line which best fitted the relationship between instantaneous firing rate and running speed. **E-F**, Only grid cells with significant phase precession in either a baseline or probe trial are included. **E**, Circular-linear correlation values relating theta phase to proportional distance through the field. **F**, The slope of the circular-linear phase precession regression line.

Supplementary Methods

Animals

13 experimentally naïve, male, ChAT-IRES-Cre (B6;129S6-Chat$^{tm2(cre)Lowl}$/J) mice, bred from a homozygous pair obtained from The Jackson Laboratory (ME, USA. Stock no: 006410), gave rise to the data presented in this paper. Mice were housed communally under a 12:12 inversed light-dark cycle with free access to food and water until surgery, at which point mice were 12-22 weeks old. Following surgery mice were held individually in Perspex cages, and after one week of recovery were food-restricted to a target weight of 85% of their pre-surgery weight. The target weight was then increased by 0.5g per week. All work was conducted within the terms of appropriate UK Home Office Project and Personal Licences.

Microdrives and surgery

The custom-built microdrives used comprised 16 HM-L coated 17µm platinum-iridium (90-10%) electrodes (California Fine Wire, CA, USA) twisted into 4 tetrodes. The tetrodes were wired to an 18-pin omnetics connector (Omnetics Connector Corp., MN, USA) and cemented to a drive-mechanism (Axona Ltd., St Albans, UK) which allowed for their advancement through turning of a trapped screw.

Anaesthesia was induced and maintained with an isoflurane-oxygen mix (1.5-3 L/min). During surgery, Viscotears Liquid Gel (carbomer) was applied to the mice's eyes to prevent corneal damage. Mice rested on a water-heated pad to maintain body temperature throughout surgery. Analgesia was provided by Carprieve (carprofen – 5mg/kg) injected subcutaneously at the start of surgery and Metacam (meloxicam – 5mg/kg) suspended in jelly administered orally once a day for 4 days following surgery.

After induction of steady anaesthesia, the animal's head was fixed in a stereotaxic frame. An incision was made along the midline to expose the skull, which was subsequently levelled in the horizontal plane. Six 0.7mm diameter screw holes were drilled in the skull, one for the ground-screw and five for screws which provided mechanical stability. Craniotomies were drilled to expose the entorhinal cortex and transverse sinus in both hemispheres for insertion of electrodes. An additional craniotomy was drilled in line with but laterally offset from the medial septum, for injection of the virus. Viruses were obtained from UNC Vector Core (NC, USA). 10 mice were injected with AAV2-hSyn-DIO-hM3D(Gq)-mCherry, and three mice were injected with the control virus AAV2-hSyn-DIO-mCherry. Viruses were injected at a rate of 100nl/min using an UMPIII Microsyringe Pump connected to a Nanofil Syringe with a 33g bevelled needle (World Precision Instruments, FL, USA). Four 300nl injections were made 0.7-0.9mm anterior to bregma, 0.8mm lateral to bregma, with an angle of 12° towards the midline, and at depths of 4.3, 3.9, 3.5 and 3.1mm ventral from the brain surface, starting at the most ventral site. The needle was held in place for 5 minutes after each injection, then moved either to the next site or removed from the brain. Ground and stabilising screws were then inserted and the electrodes moved into position over the entorhinal cortex. Four tetrodes were implanted in each hemisphere, mounted on separate drive mechanisms. Electrodes were inserted 0.3-0.5mm anterior to the anterior edge of the transverse sinus, 3.2mm lateral to lambda, with an angle of 6° in the posterior direction. The dura was removed at the insertion site and the electrodes implanted 0.8-1mm below the brain surface. A protective metal sheath was lowered around the electrodes before dental cement was applied to fix the microdrives to the skull and screws. After surgery, mice were transferred to a heated chamber until recovered from anaesthesia.

Experimental protocol and electrophysiological recording

In the second week after surgery, mice were handled and exposed to the recording environment for three to four sessions of 20 minutes, to familiarise them with the room and experimenter. The recording environment consisted of either a 90x90cm square or a 1m diameter circular enclosure, each with 50cm high walls. The enclosure was placed on a black metal ground-sheet, surrounded on one side by black curtains with a single white cue card with a desk lamp shining on it, and open on the other side to the rest of the experimental room.

At the start of the third week (i.e. at least 14 days after surgery), mice were connected via an RC-coupled unity-gain operational amplifier and a lightweight cable suspended on a pulley system to an Axona DACQ USB recording system (Axona Ltd., St Albans, UK). Each channel was amplified 9,000 to 20,00 times, bandpass filtered (360Hz – 7kHz), and recorded differentially against a reference channel on a separate tetrode. Spikes exceeding a trigger threshold (50-80uV) were sampled at 48kHz and time stamped with a 96kHz clock signal. One to four channels were also low-pass filtered (0-500Hz), either with or without a notch filter around 50Hz, and recorded continually without a reference channel at 4.8kHz and 250Hz as LFPs. Mouse location was tracked by way of an infrared LED on the operational amplifier and a camera above the enclosure recording at 50Hz. In this way, spike, LFP, and positional data were collected while the mice moved around the enclosure in search of droplets of sweetened formula baby milk scattered by the experimenter.

Each baseline or 'screening' trial lasted 15-30 minutes, terminated once the mouse was deemed to have covered the environment sufficiently to assess the spatial tuning of any recorded cells. Following recording, data was cut using an expectation-maximisation algorithm via the KlustaKwik software[1]. Putative clusters were then investigated by eye using the Waveform software (d1manson.github.io/waveform), which was used to coordinate clusters across trials and to correct for over-clustering, according to the amplitude, waveform, and temporal autocorrelation of putative cells.

In seven of the 10 hM3Dq mice, screening was continued until grid cells were recorded, with electrodes advanced 30-60µm between screening sessions, and with at least 4 hours between sessions. In three of the hM3Dq and the three control-mCherry mice, no grid cells were recorded and the experiment was commenced while large amplitude theta oscillations could be observed in the LFP. Experiments were only initiated after a minimum of three weeks following surgery to allow for virus expression.

See Supplementary Figure 1 for a pictorial representation of the experiment protocol. In the hM3Dq mice, two types of experimental day were performed. On 'single drug' days, following a baseline trial, the mouse was briefly (<30 seconds) anaesthetised with isoflurane and 3mg/kg of Clozapine-N-Oxide (CNO) or an equivalent volume of saline was injected intraperitoneally, before the mouse was returned to its home cage. That is, on single drug days, *either* CNO *or* saline was injected, with the order of the drugs across days counterbalanced between animals. One hour after the injection, a second trial was recorded, termed a 'CNO probe' or 'saline probe' trial.

In addition, four mice also had recordings made under a 'dual drug' protocol. Here, following the baseline trial, the mouse was first injected with saline, and after a one hour break, a saline probe trial was recorded. Subsequently, the mouse was then injected with CNO, and after a second one hour break, a CNO probe trial was recorded. Dual drug days thus included three trials, with the saline probe trial always leading the CNO probe trial due to the long-lasting time course of CNO action[2].

The CNO solution injected was made by dissolving 1mg of powdered CNO (Tocris Bioscience, Bristol, UK) in DMSO (Sigma-Aldrich, MO, USA) and then sterile 0.9% saline, to a final volume of 5ml with a concentration of 0.2mg/ml.

Analysis: Position and speed data

The recorded LED data was used to infer the position of the animal throughout the trial. Position data was smoothed separately in the x and y dimensions with a 400ms boxcar filter. The speed of the animal was calculated as the distance connecting the mouse's location in adjacent position samples using Pythagoras' theorem, adjusting for the pixels per metre recorded by the camera, divided by the temporal offset between samples (20ms).

Analysis: LFP

In mice where more than one LFP channel was recorded, an LFP channel from the same microdrive in which grid cells were recorded and on which large amplitude theta could be observed was used for all analyses. If no grid cells were recorded, then the LFP channel with maximal theta power was used. The same LFP channel was used for all trials in a single day.

The power in the LFP at different frequency bands was assessed by analysing the entire trial through constructing a power spectrum using Welch's method, with non-overlapping windows of 4096 samples, having down sampled the LFP from 4.8 to 1.2kHz. The power at each frequency was then log transformed. The peak theta frequency was defined as the frequency in the 6-10Hz band which had the highest power. The peak theta power was taken to be the log power of the peak in the 6-10Hz band. Theta signal to noise (S2N) was defined as the average power in a 2Hz band around the theta peak divided by the average power in the rest of the 3-25Hz frequency range.

Following Torrence & Compo 1998[3], Morlet wavelets were used to analyse changes in theta frequency as a function of running speed. 500 wavelet scales assessed the power at frequencies between 3 and 100Hz around each sample of the 250Hz LFP trace. The resulting spectrogram was then down sampled in time to match the 50Hz position sampling. The power spectra for each speed sample were then binned into 120 bins of width 0.25cm/s between 0 and 30cm/s, and the mean spectrum for each speed bin calculated. A maximum speed of 30cm/s was chosen as higher speeds were only poorly sampled. For each speed bin the frequency within a 3-11Hz window with maximum power was identified. The relationship between this frequency and the average speed in each bin was then fit with both a linear and three-term exponential functions using linear and non-linear least squares respectively. In the latter, parameter A, B, and C values were limited to the ranges 3-11, 0.5-4 and 0-1 respectively. The linear and non-linear functions fitted were defined as:

$$y = A + B * x$$

$$y = A - B * \mathrm{e}^{(-C * x)}$$

Where *x* is the animal's running speed, *y* is the frequency of maximum power in the theta window, and *A*, *B,* and *C* are parameters to be fit. In one trial (out of 95 trials overall), poor and uneven sampling of running speeds, together with the narrow speed bins, meant that there were intermediate bins with zero samples, and these trials were not analysed further.

Each model's quality of fit was assessed using the F-test:

$$F = \frac{(linRSS - expRSS)/(linDF - expDF)}{(expRSS/expDF)}$$

Where *linRSS* and *expRSS* are the residual sum of squared errors for the linear and exponential fits respectively, and *linDF* and *expDF* are the degrees of freedom of each model. Calculation of the F value enabled assessment of the significance of the difference in quality of fit between the two models, through calculation of a p-value from the F distribution.

To assess whether the apparently non-linear relationship between theta frequency and running speed was an artefact of the analysis described, we repeated the procedure on artificial sinusoidal LFP traces, whose frequency varied as a linear, exponential, or sinusoidal function of running speed. To generate the artificial LFP traces, a 50Hz-sampled vector of running speeds from a single baseline trial was linearly interpolated to 250Hz. A frequency value for each 250Hz sample was calculated, varying as a linear (*frequency = 4 + [0.15 * running-speed]),* exponential *(frequency = 8.5 – 3 * exp[-0.25 * running-speed])*, or sinusoidal (*frequency = 7.5 + 3 * [sin(running-speed)])* function of the running speed values. From the instantaneous frequency values, the phase-advance between each LFP sample was calculated, and used to construct a continuous sinusoidal trace. This artificial LFP trace was then fit with the above-described procedure, to confirm that the extracted frequency vs run-speed relationship matched the artificial data, and that the exponential fit did not outperform the linear fit even when the underlying relationship was linear or sinusoidal.

Analysis: theta modulation of single units

The entire trial was divided into contiguous blocks of position samples where the speed remained above a minimum threshold of 2cm/s. For each contiguous block with at least two spikes, a temporal autocorrelogram of length 500ms was generated from the cell's spike train. The individual autocorrelograms were then weighted according to the length of the block, with a weighted average calculated across the trial. A power spectrum of the temporal autocorrelogram was then calculated using the Fourier transform, and smoothed using a Gaussian kernel (width 187.5ms). The peak theta frequency was then defined as the frequency in the 6-10Hz band which had the highest power. Theta signal to noise (S2N) was defined as the average power in a 2Hz band around the theta peak divided by the average power in the rest of the 3-25Hz frequency range.

The significance of a cell's theta modulation was assessed using a shuffle procedure. The above procedure was repeated 100 times and the S2N calculated for artificial data in which the cell's spike times had been randomly reassigned to another time within the trial. A cell was deemed to be significantly theta modulated if its theta S2N exceeded that seen in 99/100 of the shuffles. Only cells with significant theta modulation in either a baseline or probe trial were included in hypothesis testing. Because of the small number of grid cells which showed significant theta modulation, we instead focused on cells assessed by eye as having theta-modulated temporal autocorrelograms, but which were not spatially modulated (either by the animal's location or heading direction).

The theta burst frequency of single units as a function of running speed was also assessed. To do so, the trial was first divided into two second windows, each with a one second overlap with the previous window. In each window with at least two spikes, the mean running speed and the power spectrum derived from the Fourier-transform of the spike train's temporal-autocorrelogram was calculated. The windows were then binned into 4cm/s speed bins between 0 and 24cm/s (24cm/s was chosen as the upper limit as it was the highest bin with reliable sampling of sufficient numbers of spikes), with the power spectra averaged together in each speed bin. Only bins from 0 until the highest speed bin containing >5% of the total number of valid windows were included. The frequency of maximum power in a 3-11Hz theta range was identified for each valid speed bin, and plotted as a function of the bin's average speed. The data were then fitted with linear and non-linear least squares as per the procedure described above for the LFP.

Again, as was done for the LFP, the difference in quality of the fits was assessed using the F test. Only cells which had significant theta modulation in either a baseline or probe trial, and only trials which had a significantly better ($p<0.05$) exponential fit were included in plotting and hypothesis testing. That is, in assessing the null hypothesis of no-change in the parameter values which gave the best fits following CNO administration, only cells meeting these criteria were included. These requirements aimed to restrict analysis to only those trials in which the exponential fit was sufficiently good for the best fitting parameter values to be meaningful.

Behavioural Analysis

To assess changes in the running behaviour of animals, the mean speed in both the first five minutes and across the whole trial were calculated. A distribution of speeds was also generated, as the proportion of the trial spent in 2cm/s speed bins between 0 and 30cm/s. The amount of time the animal spent sitting still (running speed < 1 cm/s) was calculated for the first five minutes of the trial.

To analyse the observed increase in avoidance of the centre of the environment, the periphery of the environment was defined as the area within 10cm of the outermost extent of position samples recorded across the entire trial. 'Perimeter dwell', a measure of avoidance of the centre, was then calculated as the proportion of the first five minutes of the trial spent within this peripheral zone.

Grid Cell Analysis

Two-dimensional firing rate maps were calculated by assigning both the animal's recorded positions and the spikes of each cell to 2x2cm bins. The firing rate in bin i was then calculated by dividing the number of spikes in a 5x5bin boxcar kernel centred on bin i by the cumulative dwell in the kernel.

The smoothed ratemap of each putative grid cell was assessed through construction of a spatial autocorrelogram, following Sargolini et al. 2006[4], in which the ratemap was shifted relative to itself and the correlation calculated at each offset. Concretely:

$$r(\tau_x, \tau_y) = \frac{n\Sigma\lambda(x,y)\lambda(x-\tau_x, y-\tau_y) - \Sigma\lambda(x,y)\Sigma\lambda(x-\tau_x, y-\tau_y)}{\sqrt{n\Sigma\lambda(x,y)^2 - \left(\Sigma\lambda(x,y)\right)^2} \cdot \sqrt{n\Sigma\lambda(x-\tau_x, y-\tau_y)^2 - \left(\Sigma\lambda(x-\tau_x, y-\tau_y)\right)^2}}$$

Where $r(\tau_x, \tau_y)$ is the autocorrelation between bins with spatial offset τ_x and τ_y. $\lambda(x,y)$ is the firing rate in bin (x,y), while n is the total number of bins. The rate map's degree of hexagonal regularity, or 'gridness', was calculated by rotating the autocorrelogram in 30° steps for 150° and taking the Pearson product-moment correlation coefficient between each rotated autocorrelogram and the un-rotated autocorrelogram, only including the area of the autocorrelogram extending from the edge of the central peak to the envelope of the six closest peaks. Gridness was defined as the maximum correlation at 30, 90 or 150° subtracted from the minimum at 60 or 120°. A cell was considered to be a grid cell if its gridness exceeded 0.3. The scale of each grid cell was also calculated, as the median distance (in cm) to the six peaks closest to the autocorrelogram's centre.

To assess the intra-trial stability each grid cell's firing pattern, each trial was divided into two halves, with a ratemap generated separately for each according to the above described procedure. A cross-correlogram was generated using the same correlation procedure discussed above, but instead using the ratemaps from the two halves of the trial rather than two copies of a single ratemap. The value at the centre of the cross-correlogram was used as a measure of the stability of the cell's firing between the two halves of the trial.

Inter-trial stability was assessed between the baseline and probe trials, again using the same procedure described above, but instead with the two ratemaps being those from the entirety of the baseline and probe trials respectively. The degree of stability was again measured as the correlation value at the centre of the cross-correlogram.

For cells to be included in hypothesis testing of gridness and stability, the cell had to have gridness > 0.3 in either a baseline or probe trial. Only trials with gridness > 0.3 were used to assess grid scale, and therefore the cells included in grid scale analyses had gridness > 0.3 in both the baseline and probe trials.

Grid Cell Speed Modulation Analysis

Following Kropff et al. 2015[5], to assess the firing rates vs speed modulation of grid cells, each cell's firing rate was calculated at the same frequency as the position sampling (50Hz) by binning spikes into 20ms bins and then smoothing with a Gaussian kernel of 60ms width and a sigma of 30ms. The regression line relating speed and firing rate was then fit for position samples above 2cm/s and below 30cm/s, with the correlation and slope identified. The correlation between speed and firing rate was termed the cell's 'speed score'.

To assess the significance of a cell's firing rate vs running speed relationship, each trial's speed score was compared to a distribution of 1000 shuffled speed scores calculated after circularly rotating the animal's speed relative to the cell's firing rate by a random temporal offset of at least 20 seconds. A grid cell was deemed to be significantly speed modulated if its speed score fell in the top 1% of the shuffled distribution of speed scores and had a gridness score above 0.3 in the same trial. Only grid cells with significant speed modulation in either a baseline or probe trial were included in hypothesis testing.

Grid Cell Phase Precession Analysis

In analysing the tendency of grid cells to display phase precession, the procedure described in Jeewajee et al. 2014[6] was followed. Briefly, the 250Hz LFP was smoothed and filtered with a Blackman filter between 6 and 10Hz. The Hilbert transform of the filtered LFP was then used to calculate the instantaneous phase of the theta oscillation for each LFP sample. The grid cell's firing rate map was produced as described above, but instead using 0.5x05cm bins and a Gaussian smoothing kernel of 100x100bins. Grid firing fields were defined using a watershed function. Fields near the edges of the environment were only included if their area exceeded 75% of the mean area of fields in the centre of the environment. The time between entry and exit from a valid firing field was termed a 'run'. Individual runs through the field were normalised so as to fall within a unit circle, and rotated so that the average movement direction of each run was left to right. The 'proportion of way along run' was then calculated as the animal's signed distance to the field peak, accounting for running direction by projecting onto the vector joining current position in the field to the edge of the field, assuming no future change in running direction. Proportion of way along run varies from -1 as the animal enters to 1 as it exits the field, with a value of 0 when crossing the field's peak. A circular-linear regression was then performed, relating proportion of way along run to the phase of the first spike in each theta cycle in the run, with slope and correlation values identified.

Only grid cells that were not modulated by heading direction were included in this analysis. Directional ratemaps were generated by assigning heading directions and spikes to 6° bins between 0 and 360°, and dividing the number of spikes by the time spent in each bin. The ratemaps were then smoothed circularly with a Gaussian smoothing kernel of 5 bins. The Kullback-Leibler divergence (KLD) comparing the cell's directional ratemap to a uniform distribution was then

calculated. A cell was considered directionally modulated and not further analysed if its KLD exceeded 0.15.

The significance of the phase precession exhibited by a grid cell was determined using a shuffling procedure. A cell was considered to display significant phase precession if its correlation value was more negative than 95% of 1000 shuffles where distance-along-run and spike-phase were randomly re-paired. To be included in hypothesis testing, a cell had to have KLD<0.15, gridness>0.3 and significant phase precession in either a baseline or probe trial.

Experimental protocol: control mice

Control mice injected with DIO-mCherry were used to assess whether any change seen in the hM3Dq mice was due to non-specific effects of CNO administration. Control mice were familiarised with the recording environment and electrophysiological recordings made as described above for hM3Dq mice.

At least three weeks after surgery, and once familiarised with the environment, three experimental days were carried out on consecutive days following the same 'dual drug' protocol used in the hM3Dq mice (see Supp Fig 1). Briefly, a baseline trial was followed by intraperitoneal injection of saline, and a one hour pause by a saline probe trial. Injection of CNO and a one hour pause then followed, before a CNO probe trial was performed. On each day, an equivalent volume of saline to the volume of CNO solution was administered, with 1mg/kg of CNO on the first day, 3mg/kg of CNO on the second day, and 5mg/kg of CNO injected on the third day.

In assessing whether any effect was seen in control mice, the measures in which a significant difference was seen in hM3Dq mice were examined by eye, averaging at the level of animal both across all drug concentrations, and separately for each drug concentration, to see if any dose-dependent effects could be observed.

Analysis: Hypothesis Testing

The analyses discussed above generated a number of measures for which we assessed whether or not there was sufficient evidence to reject the null hypothesis that there was no change in the measure following injection of CNO. Throughout this paper, these hypotheses were tested using Repeated Measures ANOVA at the level of animal. Specifically, for each measure, we averaged together values from all appropriate cells and trials to generate four values for each animal: a CNO baseline value, a saline baseline value, a CNO probe value, and a saline probe value. CNO baseline and saline baseline values were distinguished by whether the baseline trial was followed by a CNO or saline probe trial. The Repeated Measures ANOVA (RM-ANOVA) test was then used to look for an interaction effect between the baseline vs probe groups and CNO vs saline groups. That is, to assess whether the change between baseline and probe averages was significantly different between the CNO and saline groups.

The only exception to the use of the RM-ANOVA test was in analysing the inter-trial stability of grid cell firing patterns, for which a paired t-test was used, as looking at the correlation between trials meant there was only a single value for each of the CNO and saline groups.

Analysis was performed at the level of animal as cells are often not independent of one another within animals, making statistical testing problematic (e.g. for grid cells, see [7–9]). For an animal to be included in the RM-ANOVA analysis it was required to have a data point for all four groups used in statistical testing (CNO baseline, saline baseline, CNO probe, saline probe). Averaging at the level of animal therefore meant that for the LFP and behaviour analyses the statistical tests had an n of 10.

Each of the cellular analyses had further criteria for inclusion as discussed above, which lowered the number of data points. As such, some of the analyses only had a small number of data points. When no change was seen in analyses averaging at the level of animal, we therefore repeated analyses averaging at the level of cell. As instability in recordings meant that the same cell was only rarely recorded across both saline and CNO days, cells were included as long as they had a value in both CNO baseline and CNO probe, or saline baseline and saline probe trials. As such, a mixed-design ANOVA was used for hypothesis testing, to allow inclusion of cells which did not have values for all four trial types.

Immunohistochemistry and Cell Counting

At the end of the experiment, mice were anaesthetised with isoflurane before intraperitoneal injection of 0.5ml of Euthatal (sodium perntobarbital). Following loss of toe-pinch and eye-blink reflexes, mice were trans-cardially perfused with 10ml of 0.9% saline followed by 20ml of 4% paraformaldehyde (PFA). Brains were extracted and left in PFA for 4-6 hours, and then transferred to a cryoprotectant solution of 30% sucrose in tris-buffered saline (TBS) until the brains had sunk.

Brains were then cut in the coronal plane into 50µm sections using a freezing microtome. Sections were collected into TBS before being washed three times for 10 minutes in a solution of TBS and 0.3% Triton X-100. Slices were subsequently incubated with DAKO Serum-Free Protein Block for one hour. Slices were then incubated on a shaker table for 24 hours at 4°C with primary antibodies for choline acetyltransferase (ChAT) (EMD Millipore, AB144P, 1:100) and mCherry (Abcam, AB167453, 1:1000). Slices were then again washed three times in TBS with 0.3% Triton X-100, before incubation at room temperature with secondary antibodies at 1:1000 for 18 hours (ChAT: ThermoFisher Scientific, A-10042 Alexa-488 conjugated. mCherry: ThermoFisher Scientific, A-11055 Alexa-568 conjugated). Slices were washed a final three times in TBS before being mounted in Vectashield and a coverslip applied.

Slices were viewed at 2.5x and 10x magnification using an epifluorescence microscope and at 40x magnification using a Leica SP8 confocal microscope. Confocal images of different planes of the slice were layered to produce z-stacks of the entire slice. Images of the medial septum were captured using filters appropriate for the 488 and 568nm dyes conjugated to the secondary antibodies. Selected captured images were exported as TIFF files using the Leica LAS X software.

In addition, cell counting was performed manually on composite images of microscope slides at 10x magnification, captured using a Zeiss Axioscan Slide Scanner. For each animal in which cell counting was performed, the slide with the eight slices (400µm range) of the medial septum with maximal expression of mCherry were selected. Of these eight slices, cell counting was performed on one slice from each pair of adjacent slices (thus, in each animal, counting covered 4 slices over a minimum range of 300µm). The number of mCherry+, ChAT+ and mCherry+/ChAT+ cells were counted across the entire medial septal area of each slice. This enabled calculation of the proportion of ChAT+ cells co-labelled for mCherry and the proportion of mCherry+ cells co-labelled for ChAT in each slice. The values were then averaged together across the four slices counted to create average proportions for each animal.

Supplemental Methods References

1. Kadir, S. N., Goodman, D. F. M. & Harris, K. D. High-dimensional cluster analysis with the masked EM algorithm. *Neural Comput.* **26,** 2379–2394 (2014).
2. Alexander, G. M. *et al.* Remote control of neuronal activity in transgenic mice expressing evolved G protein-coupled receptors. *Neuron* **63,** 27–39 (2009).

3. Torrence, C. & Compo, G. P. A practical guide to wavelet analysis. *Bull. Am. Meteorol. Soc.* **79,** 61–78 (1998).
4. Sargolini, F. *et al.* Conjunctive representation of position, direction, and velocity in entorhinal cortex. *Science* **312,** 758–762 (2006).
5. Kropff, E., Carmichael, J. E., Moser, M.-B. & Moser, E. I. Speed cells in the medial entorhinal cortex. *Nature* **523,** 419–424 (2015).
6. Jeewajee, A. *et al.* Theta phase precession of grid and place cell firing in open environments. *Philos. Trans. R. Soc. Lond. B. Biol. Sci.* **369,** 20120532 (2014).
7. Barry, C., Hayman, R., Burgess, N. & Jeffery, K. J. Experience-dependent rescaling of entorhinal grids. *Nat. Neurosci.* **10,** 682–684 (2007).
8. Stensola, H. *et al.* The entorhinal grid map is discretized. *Nature* **492,** 72–78 (2012).
9. Yoon, K. *et al.* Specific evidence of low-dimensional continuous attractor dynamics in grid cells. *Nat. Neurosci.* **16,** 1077–1084 (2013).
